# Robust hydrophobic gold, glass and polypropylene surfaces obtained through a nanometric covalently bound organic layer†

**DOI:** 10.1039/d0ra01011a

**Published:** 2020-04-03

**Authors:** Alice Mattiuzzi, Ludovic Troian-Gautier, Jérémy Mertens, François Reniers, Jean-François Bergamini, Quentin Lenne, Corinne Lagrost, Ivan Jabin

**Affiliations:** X4C 128 Rue du Chêne Bonnet 6110 Montigny-le-Tilleul Belgium amattiuzzi@x4c.eu; Laboratoire de Chimie Organique, Université libre de Bruxelles (ULB) CP 160/06, 50 Avenue F. D. Roosevelt 1050 Brussels Belgium ijabin@ulb.be; Chemistry of Surfaces, Interfaces and Nanomaterials – ChemSIN, Université libre de Bruxelles (ULB) CP 255, Campus de la Plaine, Boulevard du Triomphe 1050 Brussels Belgium; Univ Rennes, CNRS, ISCR-UMR 6226 F-35000 Rennes France Corinne.lagrost@univ-rennes1.fr

## Abstract

The (electro)chemical grafting of a polyfluorinated calix[4]arene on gold, polypropylene and glass is reported. The modified surfaces were characterized by ellipsometry, atomic force microscopy (AFM), and by X-ray photoelectron spectroscopy (XPS). A nanometric, robust and uniform monolayer of covalently surface-bound calix[4]arenes was obtained on the three different materials. For all surfaces, contact angles higher than 110° were recorded, highlighting the hydrophobic character given by this ∼2 nm thin organic monolayer. Remarkably, the contact angle values remained unchanged after 18 months under a laboratory atmosphere. The results presented herein thus present an attractive and sustainable strategy for bringing hydrophobic properties to the interface of a wide range of materials.

## Abstract 2

The grafting of a polyfluorinated calix[4]arene-tetradiazonium derivative on various surfaces led to the formation of very robust and stable hydrophobic monolayers.

## Graphical abstract

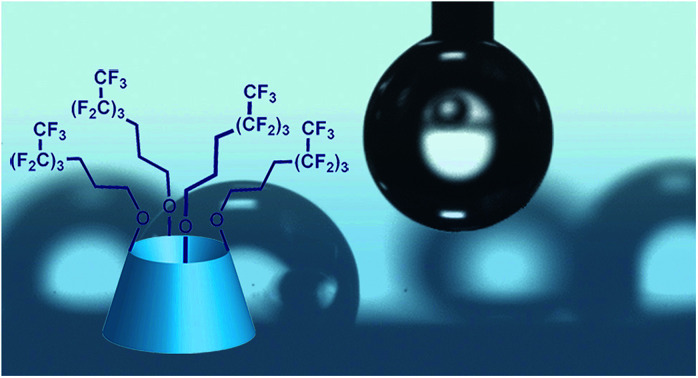

## Introduction

The development of surfaces with controlled wettability^1–3^ is of great importance for applications such as anti-biofouling,^4,5^ anti-frosting and anti-fogging,^6,7^ self-cleaning,^8^ anti-corrosion,^9,10^ or water-proofing.^11^ Wettability is usually assessed by contact angle measurements, with regimes that span from superhydrophilic, characterized by contact angles smaller than 10°, to superhydrophobic, with contact angles greater than 150° and negligible adhesion forces.^1,3,12^ Hydrophilicity and hydrophobicity are intermediate behaviours that are usually characterized by a contact angle boundary of 65°.^13,14^

An efficient approach for the development of hydrophobic surfaces consists in the grafting of polyfluorinated organic compounds bearing either a terminal thiol,^15^ phosphonic acid,^16^ silane^17^ or diazonium group,^18^ possibly combined with a roughening or a nanostructuring of the surface. In this regard, Kim *et al.* recently reported the modification of nanostructured aluminium with CF_3_(CF_2_)_9_(CH_2_)_2_–PO_3_H_2_ for anti-frosting applications.^6^ In this case, contact angles that ranged between 166.0° and 170.8° could be obtained thanks to the nanostructuring of the surface. However, derivatives with more than six CF_2_ substituents are micropollutants with deleterious environmental effects, as well as potential for bioaccumulation.^19^ Alternatives have focused on decreasing the number of CF_2_ repetitions to reduce bioaccumulation through decreased lipophilicity.^20^ Dichiarante *et al.* recently described the use of multibranched perfluoroalkyl thiol compounds and 1*H*,1*H*,2*H*,2*H*-perfluorodecanethiol to develop hydrophobic gold surfaces.^21^ Static contact angles of *ca.* 110° were measured, which marginally decreased (<10%) over two weeks. Pinson *et al.* grafted perfluoroalkyl-substituted aryl-diazonium derivatives on polymethylmethacrylate (PMMA) using hypophosphorous acid as chemical reductant.^22^ Multilayers of aryl derivatives with thickness in the range 4–10 nm were formed on PMMA, which led to static contact angles of 108°. Alternatively, stable hydrophobic surfaces with contact angles between 110° and 140° were obtained by atmospheric plasma deposition of polyfluorinated precursors.^23^

Some approaches for preparing hydrophobic surfaces require costly equipment and specially trained operator (plasma deposition, e-beam lithography, *etc*). More importantly, chemical strategies for surface modification exhibit key drawbacks. Notably, most of them are specific to a given type of materials. For example, thiols can be used only for the modification of coinage metals. Moreover, the resulting self-assembled monolayers (SAMs) show thermal and long-term stability issues, as well as non-uniform distribution across the metallic surface in the case of mixed-thiol compositions.^24,25^ Methods using silane derivatives have low reproducibility together with side reactions like polymerization.^26^ Aryldiazonium salts yield robust and stable surface coatings but with a poor control of layer thickness.^27,28^ Unless particular conditions or specifically designed diazonium derivatives are used, the formation of monolayers remains extremely challenging.^29–39^ These monolayers are nonetheless highly desired as they do not modify the bulk properties of the material but adduces specific properties.

Additionally, the formation of an organic monolayer implies the use and the presence at the surface of a very small amount of organic compounds, which is a beneficial aspect in the case of compounds with deleterious environmental effects.

**Fig. 1:**
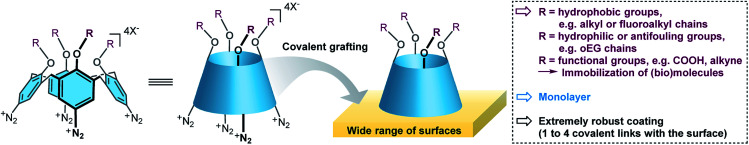
General strategy for the covalent grafting of calix[4]arene tetradiazonium derivatives.

Recently, calix[4]arene-tetradiazonium derivatives have been developed and used for (electro)chemical modifications of a wide variety of surfaces (Fig. 1).^4,40–47^ Calix[4]arenes are macrocycles composed of four phenolic units that are *para*-substituted and linked in the *ortho* position through methylene bridges. These methylene bridges prevent polymerization of the diazonium derivatives, and hence lead solely to formation of monolayers. This efficient surface modification strategy was already used on nanoparticles,^46,47^ conductive,^41–44^ semi-conductive^4^ and insulating surfaces.^45^ Importantly, the use of calix[4]arenes allows to pre-organize four reactive functions in the same direction that could yield up to four covalent bonds with the surface, and hence potentially increase the monolayer's stability. Additionally, the calix[4]arene motif can be functionalized with up to four different substituents, which yields a molecular platform that can easily be post-functionalized through standard chemical transformations such as peptide coupling or click chemistry. In the course of developing a general methodology for the preparation of thin and robust hydrophobic layers, we wanted to evaluate the grafting of a polyfluorinated calix[4]arene-tetradiazonium derivative. Note that such compounds were not described in the literature and a valuable synthetic pathway had to be developed first.

Here, we report on the synthesis of a polyfluorinated calix[4]arene-tetradiazonium derivative, 1, and its use for surface modifications of glass, gold and polypropylene (PP). This calix[4]arene derivative is decorated with four polyfluorinated chains. Due to the conductive or insulating properties of the surfaces, two grafting approaches were used, *i.e.* an electrochemical one or a chemical one. These grafting methodologies both led to the formation of a very robust hydrophobic monolayer that remained stable during 18 months.

## Results and discussion

### Synthesis of calix[4]tetra-*O*-(CH_2_)_3_C_4_F_9_ tetradiazonium 1

**Scheme 1:**
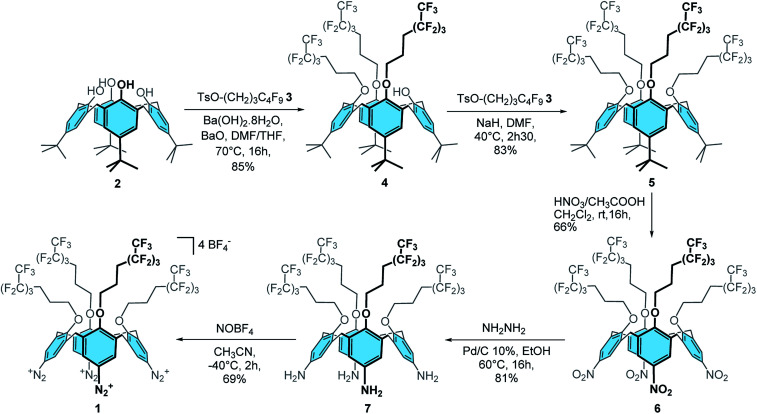
Synthesis of calix[4]tetra-*O*-(CH_2_)_3_C_4_F_9_ tetradiazonium 1.

Firstly, calix[4]tris-*O*-(CH_2_)_3_C_4_F_9_4 was obtained in 85% yield through the tris-*O*-alkylation of *p-t*Bu-calix[4]arene 2 with TsO–(CH_2_)_3_–C_4_F_9_3 (ref. 48) using Ba(OH)_2_·8H_2_O and BaO in a 1 : 1 DMF/THF mixture (Scheme 1). The tetra-substituted product 5 was then obtained in 83% yield by reaction of calixarene 4 with 3 and sodium hydride in DMF. *ipso*-Nitration with fuming nitric acid and glacial acetic acid in dichloromethane afforded compound 6 in 66% yield. Reduction of 6 with hydrazine hydrate and Pd/C 10% in ethanol at 60 °C yielded compound 7 in 81% yield. Finally, the desired calix[4]tetra-*O*-(CH_2_)_3_C_4_F_9_ tetradiazonium 1 was obtained in 69% yield through the diazotization of 7 with NOBF_4_ in acetonitrile at −40 °C. The ^1^H NMR spectrum of 1 is characteristic of a *C*_4v_ symmetrical calix[4]arene adopting a cone conformation: *i.e.* one doublet for the ArCH_2ax_ and ArCH_2eq_ protons, one broad signal for the eight CH_2_O protons and one singlet for the eight ArH protons. Besides, the downfield chemical shift of these ArH protons (*i.e.* 8.09 ppm) clearly attests of the presence of an electron-withdrawing group. Moreover, the infrared band at 2261 cm^−1^ is characteristic of the stretching of diazonium groups that, altogether, confirm the formation of 1.

### Grafting

Surface modification of glass, polypropylene and gold surfaces was performed through the *in situ* formation of diazoate salts from 1 and an aqueous NaOH solution.^45,49^ The procedure consists of soaking the cleaned surfaces for 2 h at room temperature in a 1 : 1 acetonitrile/NaOH aq (0.1 M) solution containing 1 (2 mM). Note that the presence of acetonitrile helps to solubilize the polyfluorinated calixarene species. The surfaces were then abundantly rinsed with water, acetonitrile, and exposed to ultrasonication for 10 to 30 min in dichloromethane and toluene. As a comparison, electrochemical grafting was also performed with gold substrates, by cyclic voltammetry and chronoamperometry techniques. Potential scanning between 0.5 V and −0.5 V *vs.* SCE at 100 mV s^−1^ for 5 cycles resulted in the expected progressive decrease of the current associated with the reduction of diazonium as a result of the charge-transfer blocking properties of the progressively grafted layer. Alternatively, a constant potential (−0.5 V *vs.* SCE) was applied for 10 minutes and the chronoamperogram also exhibited characteristic behaviour for electrografting of a gold interface with a sudden drop of current. In a previous work, we have shown that electrochemical and NaOH routes result in similar grafted layers.^44^

### Characterization

#### X-Ray photoelectron spectroscopy (XPS)

**Fig. 2:**
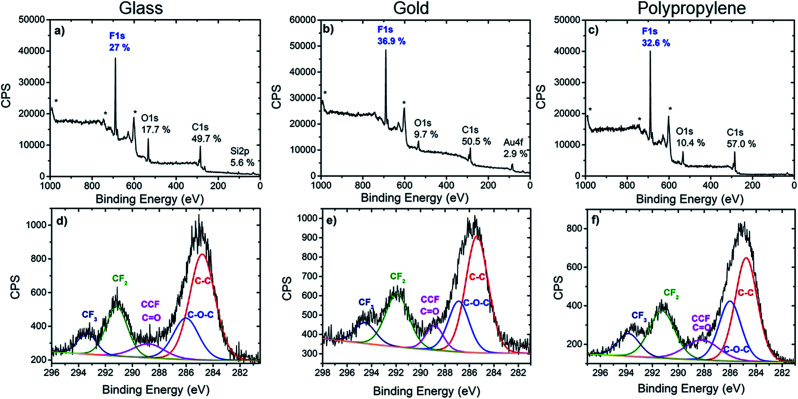
XPS survey spectra (top) and high resolution C 1s spectra (bottom) of glass (a and d), gold (b and e) and polypropylene (c and f) modified with calix[4]arene 1. * denotes Auger peaks.

XPS measurements were performed on the gold, PP and glass modified surfaces, which were extensively washed under sonication in toluene and dichloromethane prior to analyses (Fig. 2). Calix[4]arene 1 is particularly suitable for XPS measurements where the CF_2_ and CF_3_ groups are used as chemical tags. On all three surfaces, survey spectra revealed the presence of carbon C 1s (280–296 eV, 49.7–57.0% atom), oxygen O 1s (533 ± 0.4 eV, 9.7–17.7% atom), and fluorine F 1s (689.5 ± 0.7 eV, 27.0–36.9% atom). The presence of the F 1s photoelectron peak is a clear evidence of the grafting of calix[4]arene 1 on gold, polypropylene and glass. The absence of N 1s photoelectron peak at 403.8 eV (attributed to the diazonium functions) indicates the complete transformation of the diazonium cations, hence pointing to the absence of any residual adsorption of diazonium cations onto the surfaces. In addition, there is no significant contribution of N 1s photoelectrons signal, indicating that the organic layer is not bound to the surfaces through azo links as previously reported.^27^ Additionally, Au 4f_7/2_ (84.0 ± 0.3 eV, 2.9%) and Si 2p (103.1 ± 0.3 eV, 5.6%) signals were observed for the modified gold and glass surfaces, showing contributions of the metallic gold and glass substrates, respectively, to the XPS spectral signature. This observation suggests that a robust covalently-bound thin layer of calixarenes is grafted.

Further valuable information on the nature of the chemical bonds of the organic layer was extracted from the C 1s core level spectra. In all three cases, the C 1s core level spectra was decomposed into several components which reflected the chemical structure of the layer. The main component at 285 ± 0.3 eV is attributed to aryl and aliphatic C–C bonds that constitute most of the calix[4]arene backbone. Note that these C–C bonds could also arise from the surface itself in the case of polypropylene or from contamination. A component at 286.1 ± 0.3 eV is assigned to the carbon atoms involved in the C–O–C linkage present in the calix[4]arene core. The component at 288.4 ± 0.3 eV corresponds to carbon atoms in CH–CF bonds. However, these two last components could also partly contribute to oxidized contamination species.^45^ Importantly, components at 291.3 ± 0.4 eV and 293.5 ± 0.3 eV, that correspond to the CF_2_ and CF_3_ moieties present on the calix[4]arene small rim are clearly observed. The area ratio of CF_2_ and CF_3_ components is found in the range 0.35–0.40 for all substrates, in good agreement with 1 : 3 ratio expected from the molecular structure. This provides additional confirmation that the polyfluorinated calix[4]arene is indeed grafted on glass, gold and PP surfaces.

#### Atomic force microscopy (AFM)

**Fig. 3:**
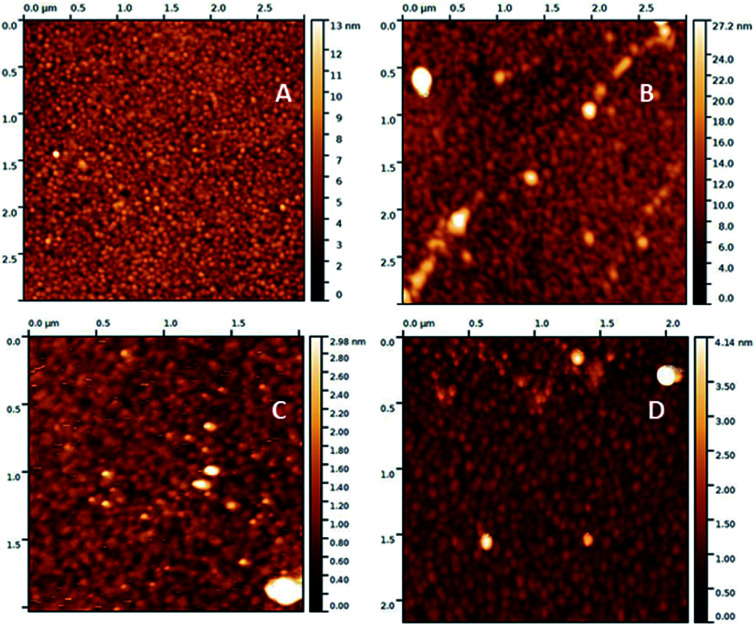
AFM topography images of (A) bare gold surface 9 μm^2^, gold surfaces after grafting of calix[4]arene 1 by (B) immersing in hydroxide solution, 9 μm^2^, (C) electrochemical reduction using CV, 4 μm^2^ and (D) electrochemical reduction using chronoamperometry, *ca.* 4 μm^2^.

AFM topography images were acquired with gold substrates modified with calix[4]arene 1 according to the two different grafting routes, *i.e.* the *in situ* formation of diazoate and the electrochemical grafting, either through cyclic voltammetry or chronoamperometry (Fig. 3). A granular surface with a classical mean roughness (rms) value about 1.3 nm was obtained for the bare gold surface. The surface roughness was minimally changed after surface modification (slight decrease), indicating that the grafted organic layers shaped to the morphology of the bare gold substrates. Such an observation is characteristic of the formation of a very thin layer. The formation of uniform and compact layers with the two methods was evidenced by the absence of accumulation or depletion zones. Due to the high roughness of the bare glass or PP substrates (rms > 8–10 nm), it was not possible to draw informative conclusions about the topography of these substrates after modification with calixarenes. However, we can conclude from the combination of XPS analyses and AFM studies that the polyfluorinated calix[4]arenes form thin films on gold, glass and polypropylene.

#### Ellipsometry

Additional information about the thickness was garnered through ellipsometry measurements on the modified gold substrates. Since this technique requires reflecting surfaces, it cannot be applied to glass and PP substrates. Again, calix[4]arene 1 was grafted following the two different routes. The height of calix[4]arene 1 (excluding the diazonium groups) was estimated to be *ca.* 1.4 nm by molecular mechanics analysis^50^ and the length of a gold–carbon bond is *ca.* 0.2 nm.^51^ Thicknesses of 2.5 ± 0.4 nm (immersion in hydroxide solution), 1.6 ± 0.1 nm (electrografting by cyclic voltammetry) and 2.9 ± 0.9 nm (electrografting by chronoamperometry) were determined. These values suggest the formation of a monolayer of calix[4]arenes on gold.

#### Contact angle measurements

**Fig. 4:**
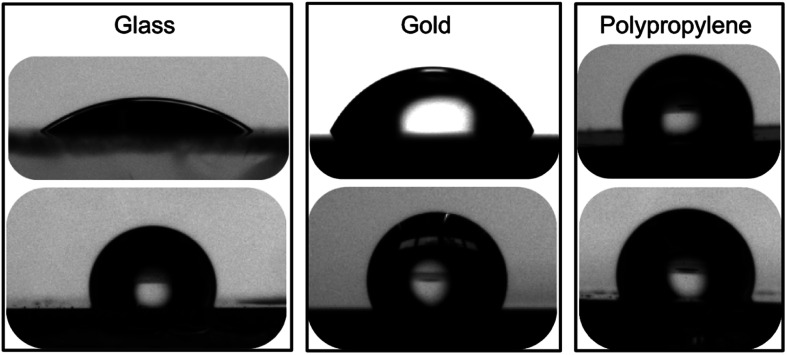
Images of 2 μL water droplets in contact with (top) bare glass, gold and polypropylene surfaces and with (bottom) the same surfaces freshly modified with calix[4]arene 1.

Initially, static contact angles of 64.7 ± 2.1°, 24.6 ± 2.0° and 102.9 ± 3.9° were determined for bare gold, glass and PP substrates, respectively (Table 1 and Fig. 4). The static contact angles were then measured with freshly modified surfaces and, in all cases, values greater than 110° were obtained, demonstrating that the grafting of calix[4]arene 1 led to considerably more hydrophobic surfaces than the initial bare surfaces (Fig. 4). Note that the contact angles were measured both on slightly and extensively washed surfaces (*i.e.* extensive rinsing with 3 different solvents under sonication for 20–30 min each) and no significant variation of the contact angle values was observed. Such a sonicating treatment represents a rather harsh work-up. Contact angle results highlight the great robustness of the grafted monolayer facing an aggressive washing treatment.

**Table 1:** Static contact angle values of bare and modified glass, gold and PP surfaces

Samples	Bare surfaces	Coated surfaces	
		Fresh	18 months-old
Glass	24.6 ± 2.0°	110.0 ± 1.8°	107.2 ± 1.7°
Gold	64.7 ± 2.1°	113.7 ± 2.2°	111.2 ± 0.7°
Polypropylene	102.9 ± 3.9°	112.6 ± 4.0°	108.1 ± 0.5°

Preliminary ageing studies were then undertaken. The coated surfaces were left on a benchtop under laboratory atmosphere for eighteen months. The surfaces were then quickly rinsed with toluene and water, and finally thoroughly dried under a stream of argon. Static contact angles of the aged surfaces were then measured. Interestingly, static contact angle values of the 18 month-old surfaces were within 5% of the values obtained for freshly modified surfaces. A control experiment with a bare gold surface that underwent the same ageing treatment led to static contact angle value of 67 ± 3.2°. These results demonstrate the remarkable robustness of the surfaces covalently modified with polyfluorinated calix[4]arene derivatives.

## Conclusions

The synthesis of a calix[4]arene tetradiazonium bearing polyfluorinated chains (1) was reported for the first time. This compound led to a covalently bound monolayer onto gold, glass and polypropylene, either through an electrochemical approach (for conductive surfaces) or through a chemical modification using sodium hydroxide (for both conductive and insulating surfaces). Both approaches gave similar results but expand this grafting methodology to a broader range of materials. The grafting was validated by ellipsometry, AFM and XPS analyses. In all cases, static contact angle values greater than 110° were determined, indicating a strong hydrophobicity despite the ultrathin layer of calixarenes that was immobilized. A remarkable aspect of this methodology was the high robustness of the polyfluorinated layer, as illustrated by the almost constant contact angle values obtained despite aggressive washing treatments and ageing studies. This grafting methodology is very simple to operate. In addition, it could be easily applied to a wide range of materials whenever a robust and thin hydrophobic coating is required.

## Experimental

### Materials and methods

All solvents and reagents were at least of reagent grade quality and were purchased either from Alfa Aesar, Sigma-Aldrich or Acros organics. 4,4,5,5,6,6,7,7,7-Nonafluoroheptanol was purchased from Fluorochem. Anhydrous DMF and CHCl_3_ were obtained from Acros organics. Gold coated silicon wafer (1000 Å layer thickness) and TBAPF_6_ (electrochemical grade) were purchased from Sigma-Aldrich. Polypropylene (biaxially oriented, 50 μm) was purchased from Goodfellow. The typical size of the surfaces reported in this paper was 1 cm^2^. Ultrapure water was obtained *via* a Millipore Milli-Q system (18.2 MΩ.cm).

**Caution!** Although we have not encountered any problem, it is noted that diazonium salts are potentially explosive and should be handled with appropriate precautions.

### Electrochemical grafting

Gold surfaces were first immersed in a “piranha” solution (H_2_SO_4_/H_2_O_2_ 3 : 1) and sonicated for 10 minutes. **Caution!** This solution is a very strong oxidant and should be handled very carefully. They were then washed with concentrated H_2_SO_4_ and with ultrapure water and dried under argon atmosphere. The electrochemical grafting was performed by cyclic voltammetry (CV) or chronoamperometry (CA) in 0.1 M TBAPF_6_ anhydrous acetonitrile containing 1 mM of calix[4]arene-tetradiazonium salt using an Autolab PGSTAT 30 potentiostat/galvanostat (Metrohm Autolab B.V.). For CV, the potential was scanned between +0.5 V and −0.5 V *vs.* SCE at 100 mV s^−1^ (5 cycles). For CA, a constant potential of −0.5 V *vs.* SCE was applied for 10 min. An extensive rinsing with solvents of decreasing polarity (acetonitrile, dichloromethane, toluene) under sonication for 20–30 min was necessary to remove the accumulation of loosely adsorbed calix[4]arenes at the top of the covalently grafted layer. This accumulation of adsorbed material was probably due to strong interactions between the polyfluorinated chains. The surfaces were finally dried using an argon flow.

### Chemical grafting using sodium hydroxide

Gold and glass surfaces were first immersed in a “piranha” solution (H_2_SO_4_/H_2_O_2_ 3 : 1) and sonicated for 10 min. Gold surfaces were then washed with concentrated H_2_SO_4_ and with ultrapure water and dried under argon atmosphere. Glass surfaces were then thoroughly rinsed with ultrapure water and cleaned under sonication for 10 min in absolute ethanol, acetone, isopropanol and ultrapure water before being dried under argon atmosphere. Polypropylene surfaces were cleaned with isopropanol under sonication for 10 min, washed with ultrapure water and dried using an argon flow. The surfaces were then immersed in a 2 mM suspension of the diazonium salt in a 1 : 1 mixture of acetonitrile and aqueous 0.1 M sodium hydroxide. The surfaces were left soaking for 2 h without stirring in order to avoid any mechanical friction of the surface with the magnetic stirrer. An extensive rinsing with solvents of decreasing polarity (acetonitrile, dichloromethane, toluene) under sonication for 20–30 min was necessary to remove the accumulation of loosely adsorbed calix[4]arenes at the top of the covalently grafted layer. This accumulation of adsorbed material was probably due to strong interactions between the polyfluorinated chains. The surfaces were finally dried using an argon flow.

### Atomic force microscopy (AFM)

AFM experiments were performed with a NT-MDT Ntegra microscope. Topography images were acquired in semi-contact mode using silicon nitride tips with a resonance frequency of about 350 kHz.

### Ellipsometry measurements

The thicknesses of the layers deposited on gold were measured using a spectroscopic a-SE ellipsometer (J. A. Woollam, Co.). The polarization angles *Ψ* and *Δ* were recorded in the 380–900 nm wavelength range at different incident angles, 65°, 70° and 75°. Prior to modification, *Ψ* and *Δ* were measured to build a substrate optical model; the refractive index (*n*) and the extinction coefficient (*k*) were determined as 0.48 and 3.54, respectively. This model was used after modification to consider the substrate's contribution in the ellipsometry measurements. The thickness of the organic layer was estimated through a fitting according to the Cauchy model with *n* = 1.5 and *k* = 0. The validity of the fitting was quantified by the mean square error (MSE), with value below 4. All the values given in the manuscript are averaged values of 3–5 measurements performed on four different substrates.

### XPS measurements

XPS analysis was performed on a Physical Electronics PHI-5600 photoelectron spectrometer. Survey scans were used to determine the elemental chemical composition of the surface. Narrow-region photoelectron spectra were used for the chemical study of the C 1s. The spectra were acquired using the Mg anode (1253.6 eV) operating at 300 W. Wide surveys were acquired at a pass-energy of 187.5 eV with a five-scans accumulation (time per step: 50 ms, eV per step: 0.8) and high-resolution spectra of the C 1s peaks were recorded at a pass-energy of 23.5 eV with an accumulation of 5 scans (time per step: 150 ms, eV per step: 0.05). Spectral calibration was determined by setting C 1s at 285 eV. The atomic concentration for surface composition was estimated using the integrated peaks areas. The peaks areas were normalized by the manufacturer-supplied sensitivity factor (*S*_C 1s_ = 0.205, *S*_F 1s_ = 1, *S*_O 1s_ = 0.63, *S*_N 1s_ = 0.38, *S*_Si 2p_ = 0.17, *S*_Au 4f_ = 3.32). The core level C 1s spectra were peak-fitted using the CasaXPS software (Casa Software, Ltd., version 2.3.16).

### Contact angles measurements

The static contact angles of 2 μL Milli-Q water drops were measured on five different spots and two or three different samples with an easy drop goniometer (Krüss). The contact angles were determined using tangent 1 circle fitting, or Young–Laplace fitting models.

### Synthetic procedures

#### Synthesis of 4,4,5,5,6,6,7,7,7-nonafluoroheptyl 4-methylbenzenesulfonate 3

4,4,5,5,6,6,7,7,7-Nonafluoroheptanol (25 g, 90 mmol), triethylamine (17 mL, 134 mmol), *p*-toluenesulfonyl chloride (16.1 g, 84 mmol) and 4-dimethylaminopyridine (0.4 g, 3.1 mmol) were solubilized in 250 mL of dichloromethane. The reaction mixture was stirred at room temperature for 4 h 30 min after which 50 mL of 2 M HCl were added. The organic layer was then washed with water until the aqueous layer reached a pH of 7. The organic layer was then evaporated under reduced pressure and the resulting residue was purified by column chromatography (SiO_2_, C_6_H_12_/CH_2_Cl_2_, 7 : 3), affording compound 3 as a colourless liquid (29.3 g, 68 mmol, 81%). ^1^H NMR (300 MHz, CDCl_3_, 298 K): *δ*_(ppm)_ = 1.91–2.00 (m, 2H, C*H*_2_CH_2_C_4_F_9_), 2.05–2.21 (m, 2H, C*H*_2_C_4_F_9_), 2.45 (s, 3H, C*H*_3_Ts), 4.11 (t, ^3^*J* = 6.0 Hz, 2H, TsOC*H*_2_), 7.36 (d, ^3^*J* = 8.3 Hz, 2H, ArH), 7.79 (d, ^3^*J* = 8.3 Hz, 2H, ArH). The ^1^H NMR spectrum of 3 is in accordance with the one reported in the literature.^48^

#### Synthesis of calix[4]tris-*O*-(CH_2_)_3_C_4_F_9_4

*p-t*Bu-calix[4]arene 2 (6.0 g, 9.2 mmol) was suspended in 150 mL of a 1 : 1 DMF/THF mixture. Ba(OH)_2_·8H_2_O (9.3 g, 29 mmol) and BaO (8.9 g, 65 mmol) were then added. The reaction mixture was stirred at room temperature and TsO-(CH_2_)_3_C_4_F_9_3 (25.9 g, 60 mmol) was added. The mixture was then heated at 70 °C for 14 h. The mixture was evaporated under reduced pressure and the resulting residue was dissolved in 450 mL of dichloromethane. The organic layer was washed once with 100 mL of 2 M HCl and then with water until the aqueous layer reached a pH of 7. The organic layer was then evaporated under reduced pressure to yield compound 4 as a yellow solid (11.1 g, 7.8 mmol, 85%). Mp = 60 °C. IR (NaCl) *ν* (cm^−1^) = 2963, 1642, 1481, 1228, 1134, 1016. ^1^H NMR (400 MHz, CDCl_3_, 298 K): *δ*_(ppm)_ = 0.82 (s, 18H, *t*Bu), 1.33 (s, 9H, *t*Bu), 1.34 (s, 9H, *t*Bu), 2.10–2.60 (m, 12H, C*H*_2_C*H*_2_C_4_F_9_), 3.23 (d, ^2^*J* = 12.4 Hz, 2H, ArC*H*_2eq_), 3.27 (d, ^2^*J* = 13.2 Hz, 2H, ArC*H*_2eq_), 3.89 (t, ^3^*J* = 6.4 Hz, 4H, OC*H*_2_), 3.99 (t, ^3^*J* = 8.0 Hz, 2H, OCH_2_), 4.23 (d, ^2^*J* = 12.8 Hz, 2H, ArC*H*_2ax_), 4.27 (d, ^2^*J* = 12.4 Hz, 2H, ArC*H*_2ax_), 5.17 (s, 1H, OH), 6.52 (mult., 4H, ArH), 7.08 (s, 2H, ArH), 7.17 (s, 2H, ArH). ^13^C NMR (100 MHz, CDCl_3_, 298 K): *δ*_(ppm)_ = 20.9, 21.2, 27.4 (t, ^3^*J* = 21.3 Hz), 28.1 (t, ^3^*J* = 22.4 Hz), 31.1, 31.2, 31.4, 31.7, 31.8, 33.8, 34.0, 34.3, 73.2, 74.7, 124.9, 125.2, 125.3, 126.1, 129.4, 131.6, 131.8, 135.7, 142.0, 145.8, 146.4, 150.6, 151.2, 153.3. HRMS (ESI^+^): *m*/*z* calcd for C_65_H_75_F_27_NO_4_ [M + NH_4_]^+^: 1446.5294; found: 1446.5259.

#### Synthesis of calix[4]tetra-*O*-(CH_2_)_3_C_4_F_9_5

Calix[4]tris-*O*-(CH_2_)_3_C_4_F_9_4 (5.0 g, 3.5 mmol) was suspended in 120 mL of anhydrous DMF. Sodium hydride (60% in mineral oil, 861 mg, 22 mmol) was then added in one portion. The mixture was stirred for 10 minutes after which TsO-(CH_2_)_3_C_4_F_9_3 (3.2 g, 7.3 mmol) was added and the mixture was heated at 40 °C for 2 h 30 min. The solvent was removed under reduced pressure and the resulting residue was dissolved in 500 mL of dichloromethane. The organic layer was washed four times with 200 mL of water. The organic layer was evaporated under reduced pressure and the residue was purified by column chromatography (SiO_2_, C_6_H_12_/CH_2_Cl_2_, 9 : 1), affording compound 5 as a white solid (4.9 g, 2.9 mmol, 83%). Mp = 121 °C. IR (NaCl) *ν* (cm^−1^) = 2962, 1643, 1480, 1228, 1134. ^1^H NMR (400 MHz, CDCl_3_, 298 K): *δ*_(ppm)_ = 1.09 (s, 36H, *t*Bu), 2.03–2.36 (m, 16H, C*H*_2_C*H*_2_C_4_F_9_), 2.19 (d, ^2^*J* = 12.8 Hz, 4H, ArC*H*_2eq_), 3.91 (t, ^3^*J* = 7.6 Hz, 8H, OC*H*_2_), 4.29 (d, ^2^*J* = 12.4 Hz, 4H, ArC*H*_2ax_), 6.81 (s, 8H, ArH). ^13^C NMR (100 MHz, CDCl_3_, 298 K): *δ*_(ppm)_ = 21.1, 27.5 (t, ^3^*J* = 23.1 Hz), 31.2, 31.5, 34.0, 74.1, 125.4, 133.6, 145.4, 153.0. HRMS (ESI^+^): *m*/*z* calcd for C_72_H_80_F_36_NO_4_ [M + NH_4_]+: 1706.5487; found: 1706.5507.

#### Synthesis of calix[4]tetra-*O*-(CH_2_)_3_C_4_F_9_ tetra-NO_2_6

Calix[4]tetra-*O*-(CH_2_)_3_C_4_F_9_5 (4.9 g, 3 mmol) was solubilized in 200 mL of dichloromethane. 40 mL of a 1 : 1 mixture of fuming nitric acid and glacial acetic acid were then added. The reaction mixture was stirred at room temperature overnight. The mixture was then evaporated under reduced pressure. The residue was solubilized in 500 mL of dichloromethane and extracted with water until the aqueous phase reached a pH of 7. The organic layer was then evaporated under reduced pressure. The residue was dissolved in methanol, filtered and the filtrate was evaporated under reduced pressure to yield compound 6 as a yellow solid (3.2 g, 1.9 mmol, 66%). Mp = 160 °C (dec.). IR (NaCl) *ν* (cm^−1^) = 2965, 1642, 1527, 1349, 1227, 1132, 1007. ^1^H NMR (400 MHz, CDCl_3_, 298 K): *δ*_(ppm)_ = 2.03–2.27 (m, 16H, C*H*_2_C*H*_2_C_4_F_9_), 3.51 (d, ^2^*J* = 14.0 Hz, 4H, ArC*H*_2eq_), 4.06 (t, ^3^*J* = 7.2 Hz, 8H, OC*H*_2_), 4.43 (d, ^2^*J* = 14.0 Hz, 4H, ArC*H*_2ax_), 7.62 (s, 8H, ArH). ^13^C NMR (100 MHz, CDCl_3_, 298 K): *δ*_(ppm)_ = 21.3, 27.2 (t, ^3^*J* = 22.9 Hz), 31.2, 74.8, 124.5, 135.3, 143.7, 160.6. This compound could not be detected by ESI-HRMS.

#### Synthesis of Calix[4]tetraaniline tetra-*O*-(CH_2_)_3_C_4_F_9_7

Calix[4]tetra-*O*-(CH_2_)_3_C_4_F_9_ tetra-NO_2_6 (3.2 g, 1.9 mmol) was suspended in 30 mL of ethanol. Pd/C 10% (0.15 g) was then added followed by hydrazine hydrate (10 mL, 206 mmol). The reaction mixture was heated at 60 °C overnight. The mixture was then filtered on Celite that was further washed with ethanol and dichloromethane. The filtrate was then evaporated under reduced pressure to yield compound 7 as a pale-yellow solid (2.4 g, 1.5 mmol, 81%). Mp = 131 °C. IR (NaCl) *ν* (cm^−1^) = 2968, 1624, 1470, 1224, 1133, 1013. ^1^H NMR (400 MHz, CDCl_3_, 298 K): *δ*_(ppm)_ = 1.92–2.23 (m, 16H, C*H*_2_C*H*_2_C_4_F_9_), 2.80 (bs, 8H, N*H*_2_), 2.99 (d, ^2^*J* = 13.6 Hz, 4H, ArC*H*_2eq_), 3.81 (t, ^3^*J* = 7.6 Hz, 8H, OC*H*_2_), 4.18 (d, ^2^*J* = 13.6 Hz, 4H, ArC*H*_2ax_), 6.08 (s, 8H, ArH). ^13^C NMR (100 MHz, CDCl_3_, 298 K): *δ*_(ppm)_ = 21.9, 27.5 (t, ^3^*J* = 23.2 Hz), 31.1, 73.8, 116.1, 135.3, 141.0, 149.2. HRMS (ESI^+^): *m*/*z* calcd for C_56_H_49_F_36_N_4_O_4_ [M + H]^+^: 1525.3173; found: 1525.3202.

#### Synthesis of calix[4]tetra-*O*-(CH_2_)_3_C_4_F_9_ tetradiazonium 1

Calix[4]tetraaniline tetra-*O*-(CH_2_)_3_C_4_F_9_7 (99 mg, 0.065 mmol) was dissolved in 1 mL of acetonitrile. The mixture was cooled at −40 °C and NOBF_4_ (42 mg, 0.360 mmol) was added in one portion. The reaction mixture was stirred at −40 °C under argon for two hours. The mixture was then concentrated under reduced pressure and the resulting residue was washed twice with 1 mL of diethyl ether and twice with 1 mL of absolute ethanol, affording product 1 in 69% yield. IR (NaCl) *ν* (cm^−1^) = 3069, 2987, 2261, 1570, 1226, 1133, 1030. ^1^H NMR (400 MHz, CD_3_CN, 298 K): *δ*_(ppm)_ = 2.13–2.48 (m, 16H, C*H*_2_C*H*_2_C_4_F_9_), 3.83 (d, ^2^*J* = 14.4 Hz, 4H, ArC*H*_2eq_), 4.25 (m, 8H, OC*H*_2_), 4.49 (d, ^2^*J* = 14.4 Hz, 4H, ArC*H*_2ax_), 8.09 (s, 8H, ArH). ^13^C NMR (100 MHz, CDCl_3_, 298 K): *δ*_(ppm)_ = 22.0, 27.3 (t, ^3^*J* = 22.0 Hz), 30.9, 77.2, 109.3, 135.5, 138.5, 167.2.

## Conflicts of interest

L. T.-G. was a postdoctoral researcher for X4C between October 2014 and September 2015. I. J. and C. L. are shareholders of X4C. I. J. is a consultant for X4C. All other authors declare that they have no conflict of interest.

## Supplementary Material

RA-010-D0RA01011A-s001
