# Supplementary material for: Robust hydrophobic gold, glass and polypropylene surfaces obtained through a nanometric covalently bound organic layer
Source: RSC Adv. 2020 Apr 3;10(23):13553–61. doi: 10.1039/d0ra01011a (PMC9051540; doi:10.1039/d0ra01011a)
Supplement: RA-010-D0RA01011A-s001 [file RA-010-D0RA01011A-s001.pdf]

Electronic Supplementary Material (ESI) for RSC Advances.
This journal is © The Royal Society of Chemistry 2020

Supplementary Information

Robust Hydrophobic Gold, Glass and Polypropylene Surfaces Obtained Through a Nanometric Covalently Bound Organic Layer

Alice Mattiuzzi,[⊥]* Ludovic Troian-Gautier,[†] Jérémy Mertens,[‡] François Reniers,[‡] Jean-François Bergamini,[£] Quentin Lenne,[£] Corinne Lagrost[£]* and Ivan Jabin[†]*

[⊥]X4C, 128 Rue du chêne Bonnet, 6110 Montigny-le-Tilleul, Belgium

[†] Laboratoire de Chimie Organique, Université Libre de Bruxelles (ULB), CP 160/06, 50 avenue F.D. Roosevelt, 1050 Brussels, Belgium.

[£] Univ Rennes, CNRS, ISCR-UMR 6226, F-35000 Rennes (France)

[‡] Chemistry of Surfaces, Interfaces and Nanomaterials – ChemSIN, Université Libre de Bruxelles (ULB), CP 255, Campus de la Plaine, boulevard du Triomphe, 1050 Brussels, Belgium.

Table of Contents

GENERAL INFORMATION S3

EXPERIMENTAL SECTION **S3**

FIGURE S1. AFM TOPOGRAPHY OF GLASS SURFACES, LEFT 10 µM X 10 µM, RIGHT 30 µM X30 µM........ S3

FIGURE S2. ELLIPSOMETRIC ANGLES Ψ (PSI) AND Δ (DELTA) AS A FUNCTION OF THE WAVELENGTH AT DIFFERENT INCIDENT ANGLES, 65°, 70° AND 75° FOR BARE GOLD SURFACE. CONTINUOUS LINES REPRESENT THE ACQUIRED DATA AND THE DOTTED BLACK LINES CORRESPOND TO THE CAUCHY FIT. S3

FIGURE S3. ELLIPSOMETRIC ANGLES Ψ (PSI) AND Δ (DELTA) AS A FUNCTION OF THE WAVELENGTH AT DIFFERENT INCIDENT ANGLES, 65°, 70° AND 75° FOR A GOLD SURFACE MODIFIED WITH CALIX[4]ARENE **1** THROUGH DIAZOATE ROUTE IN NAOH. CONTINUOUS LINES REPRESENT THE ACQUIRED DATA AND THE DOTTED BLACK LINES CORRESPOND TO THE CAUCHY FIT........ S4

FIGURE S4.ELLIPSOMETRIC ANGLES Ψ (PSI) AND Δ (DELTA) AS A FUNCTION OF THE WAVELENGTH AT DIFFERENT INCIDENT ANGLES, 65°, 70° AND 75° FOR A GOLD SURFACE MODIFIED WITH CALIX[4]ARENE **1** THROUGH ELECTROCHEMICAL ROUTE BY CA. CONTINUOUS LINES REPRESENT THE ACQUIRED DATA AND THE DOTTED BLACK LINES CORRESPOND TO THE CAUCHY FIT........ S4

FIGURE S5. ELLIPSOMETRIC ANGLES Ψ (PSI) AND Δ (DELTA) AS A FUNCTION OF THE WAVELENGTH AT DIFFERENT INCIDENT ANGLES, 65°, 70° AND 75° FOR A GOLD SURFACE MODIFIED WITH CALIX[4]ARENE **1** THROUGH ELECTROCHEMICAL ROUTE BY CV. CONTINUOUS LINES REPRESENT THE ACQUIRED DATA AND THE DOTTED BLACK LINES CORRESPOND TO THE CAUCHY FIT........ S5

FIGURE S6. ^{1}H NMR (298K, 400MHZ) SPECTRUM OF **3** IN $CDCl_3$. S = SOLVENT AND W = WATER........ S6

FIGURE S7.^{1}H NMR (298K, 400MHZ) SPECTRUM OF **4** IN $CDCl_3$. S = SOLVENT AND W = WATER S6

FIGURE S8. ^{13}C NMR (298K, 100MHZ) SPECTRUM OF **4** IN $CDCl_3$. S = SOLVENT........ S6

FIGURE S9. COSY NMR (298K, 400MHZ) SPECTRUM OF **4** IN $CDCl_3$. S7

FIGURE S10. HSQC NMR (298K, 400MHZ) SPECTRUM OF **4** IN $CDCl_3$. S7

FIGURE S11. HMBC NMR (298K, 400MHZ) SPECTRUM OF **4** IN $CDCl_3$. S8

FIGURE S12. ^{1}H NMR (298K, 400MHZ) SPECTRUM OF **5** IN $CDCl_3$. S = SOLVENT AND W = WATER........ S8

FIGURE S13. ^{13}C NMR (298K, 100MHZ) SPECTRUM OF **5** IN $CDCl_3$. S = SOLVENT........ S9

FIGURE S14. COSY NMR (298K, 400MHZ) SPECTRUM OF **5** IN $CDCl_3$. S9

FIGURE S15. HSQC NMR (298K, 400MHZ) SPECTRUM OF **5** IN $CDCl_3$. S10

FIGURE S16. HMBC NMR (298K, 400MHZ) SPECTRUM OF **5** IN $CDCl_3$. S10

FIGURE S17. ^{1}H NMR (298K, 400MHZ) SPECTRUM OF **6** IN $CDCl_3$. S = SOLVENT, W = WATER AND G = GREASE........ S11

FIGURE S18. ^{13}C NMR (298K, 100MHZ) SPECTRUM OF **6** IN $CDCl_3$. S = SOLVENT AND G = GREASE........ S11

FIGURE S19. COSY NMR (298K, 400MHZ) SPECTRUM OF **6** IN $CDCl_3$. S12

FIGURE S20. HSQC NMR (298K, 400MHZ) SPECTRUM OF **6** IN $CDCl_3$. S12

FIGURE S21. HMBC NMR (298K, 400MHZ) SPECTRUM OF **6** IN $CDCl_3$. S13

FIGURE S22. ^{1}H NMR (298K, 400MHZ) SPECTRUM OF **7** IN $CDCl_3$. S = SOLVENT........ S13

FIGURE S23. ^{13}C NMR (298K, 100MHZ) SPECTRUM OF **7** IN $CDCl_3$. S = SOLVENT........ S14

FIGURE S24. COSY NMR (298K, 400MHZ) SPECTRUM OF **7** IN $CDCl_3$. S14

FIGURE S25. HSQC NMR (298K, 400MHZ) SPECTRUM OF **7** IN $CDCl_3$. S15

FIGURE S26. HMBC NMR (298K, 400MHZ) SPECTRUM OF **7** IN $CDCl_3$. S15

FIGURE S27. ^{1}H NMR (298K, 400MHZ) SPECTRUM OF **1** IN CD_3CN. S = SOLVENT, W = WATER. S16

FIGURE S28. ^{13}C NMR (298K, 100MHZ) SPECTRUM OF **1** IN CD_3CN. S = SOLVENT. S16

FIGURE S29. COSY NMR (298K, 400MHZ) SPECTRUM OF **1** IN CD_3CN. S17

FIGURE S30. HSQC NMR (298K, 400MHZ) SPECTRUM OF **1** IN CD_3CN. S17

FIGURE S31. HMBC NMR (298K, 400MHZ) SPECTRUM OF **1** IN CD_3CN. S18

General Information: The ^{1}H NMR, ^{13}C NMR and 2D NMR were performed with a Varian-400 VNMRJ System. The chemical shifts are expressed in ppm and determined in comparison of the deuterated solvent used as internal reference. Most of ^{1}H NMR signals were attributed through 2D NMR analyses (COSY, HSQC). Abbreviation: s: singlet, d: doublet, t: triplet, m: massif, mult: multiplet.

Experimental section:

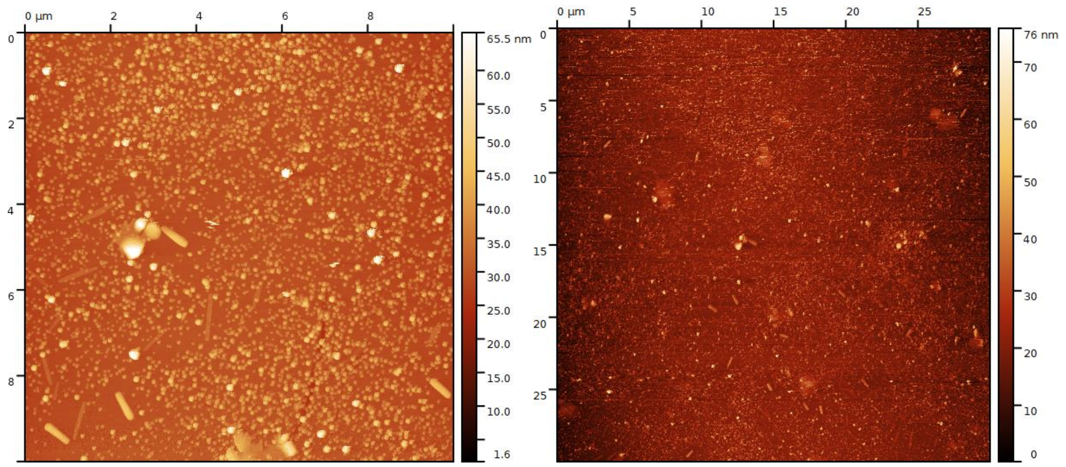

Figure S1. AFM topography of glass surfaces, left 10 µm x 10 µm, right 30 µm x30 µm

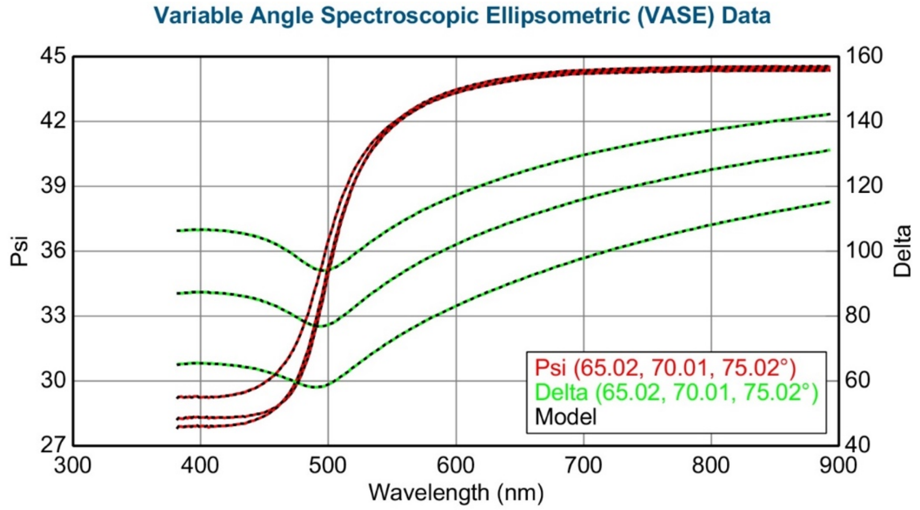

Figure S2. Ellipsometric angles ψ (Psi) and Δ (Delta) as a function of the wavelength at different incident angles, 65°, 70° and 75° for bare gold surface. Continuous lines represent the acquired data and the dotted black lines correspond to the Cauchy fit.

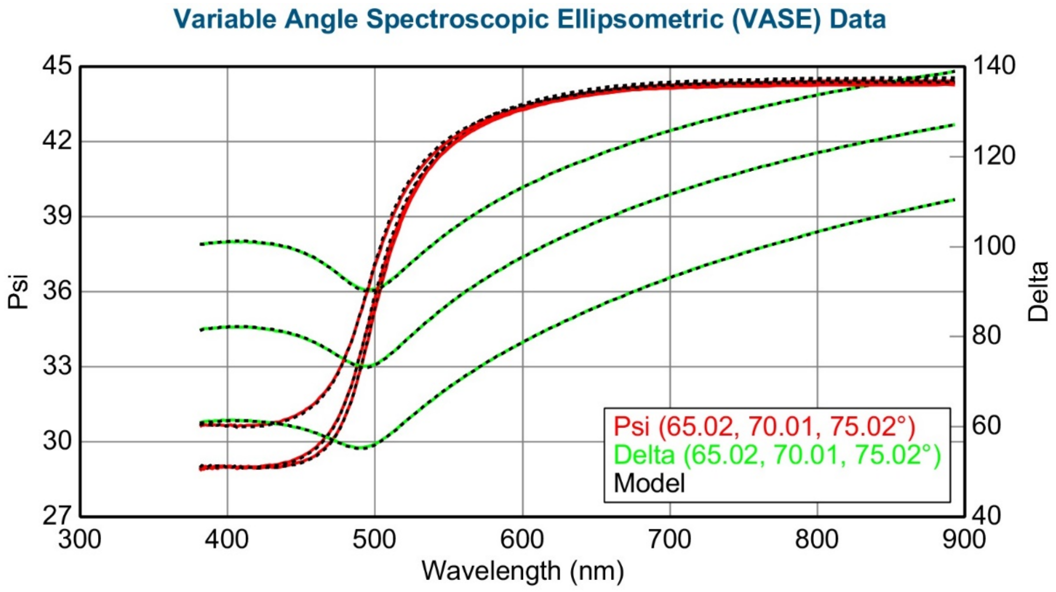

Figure S3. Ellipsometric angles ψ (Psi) and Δ (Delta) as a function of the wavelength at different incident angles, 65°, 70° and 75° for a gold surface modified with calix[4]arene **1** through diazoate route in NaOH. Continuous lines represent the acquired data and the dotted black lines correspond to the Cauchy fit.

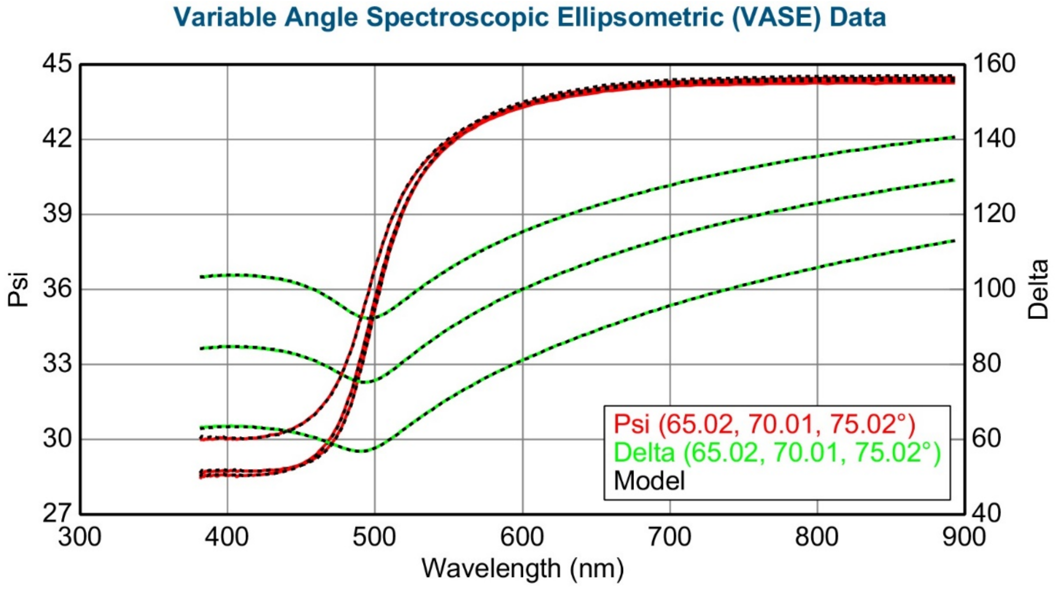

Figure S4.Ellipsometric angles ψ (Psi) and Δ (Delta) as a function of the wavelength at different incident angles, 65°, 70° and 75° for a gold surface modified with calix[4]arene **1** through electrochemical route by CA. Continuous lines represent the acquired data and the dotted black lines correspond to the Cauchy fit.

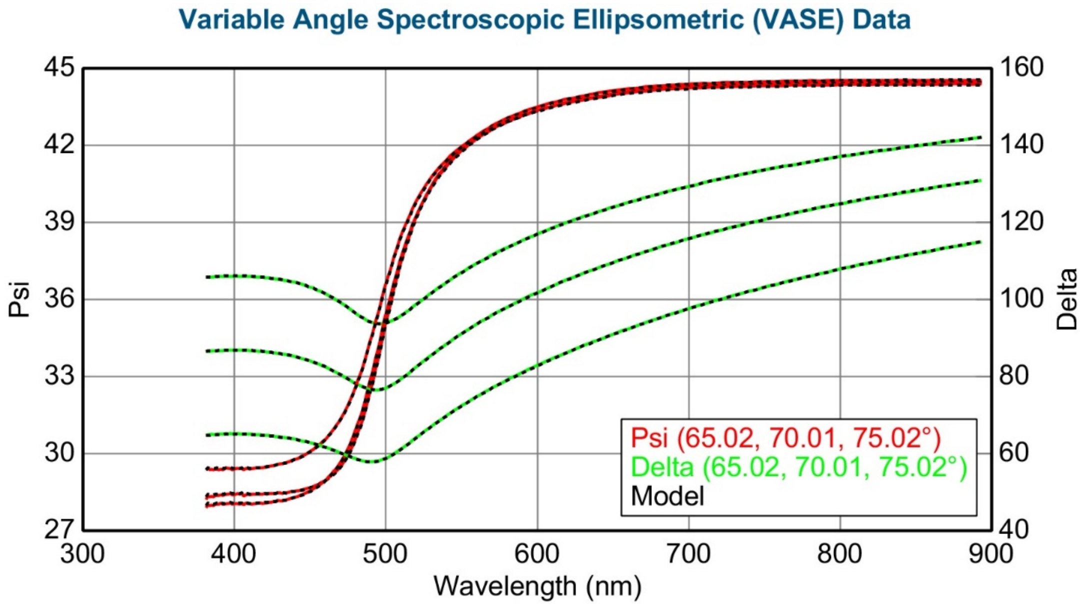

Figure S5. Ellipsometric angles ψ (Psi) and Δ (Delta) as a function of the wavelength at different incident angles, 65°, 70° and 75° for a gold surface modified with calix[4]arene **1** through electrochemical route by CV. Continuous lines represent the acquired data and the dotted black lines correspond to the Cauchy fit.

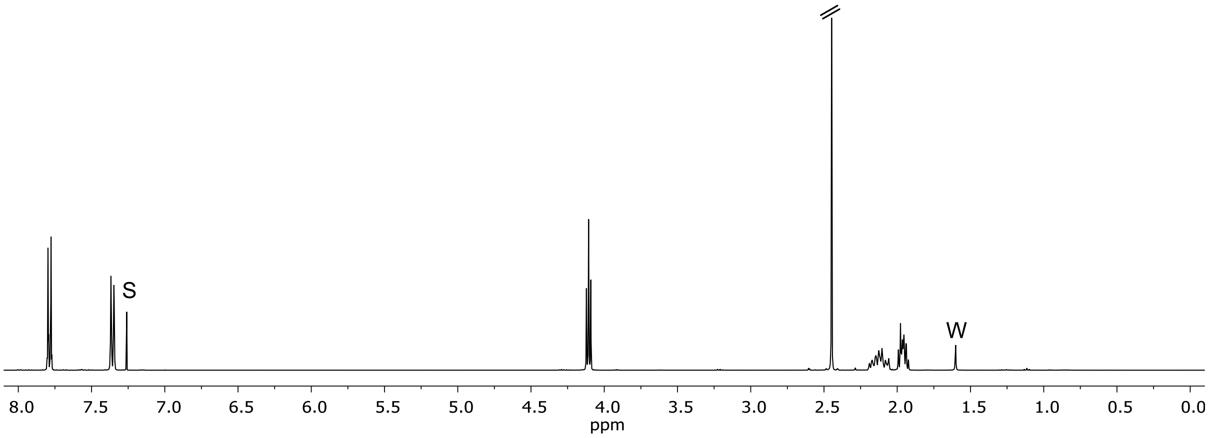

Figure S6. ^{1}H NMR (298K, 400MHz) spectrum of **3** in $CDCl_3$. S = solvent and W = water

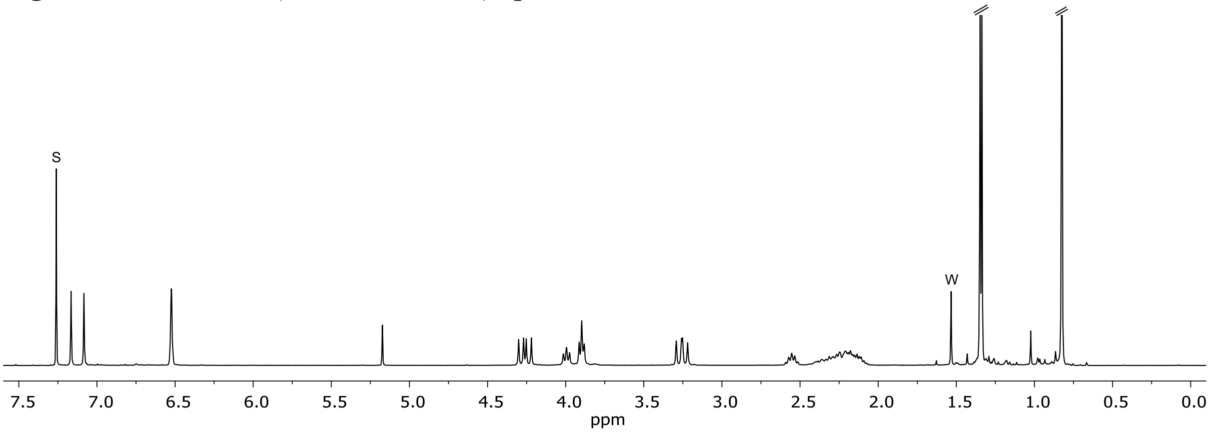

Figure S7.^{1}H NMR (298K, 400MHz) spectrum of **4** in $CDCl_3$. S = solvent and W = water

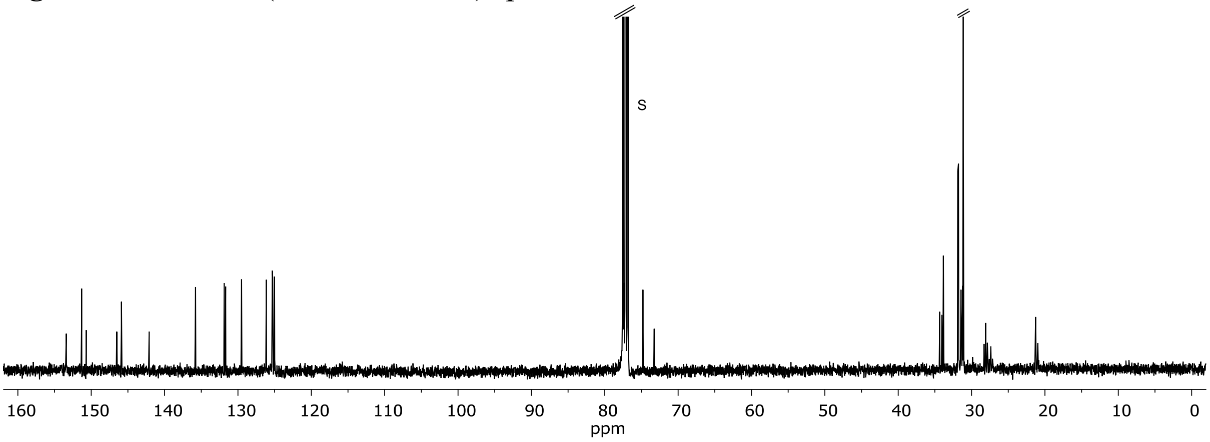

Figure S8. ^{13}C NMR (298K, 100MHz) spectrum of **4** in $CDCl_3$. S = solvent

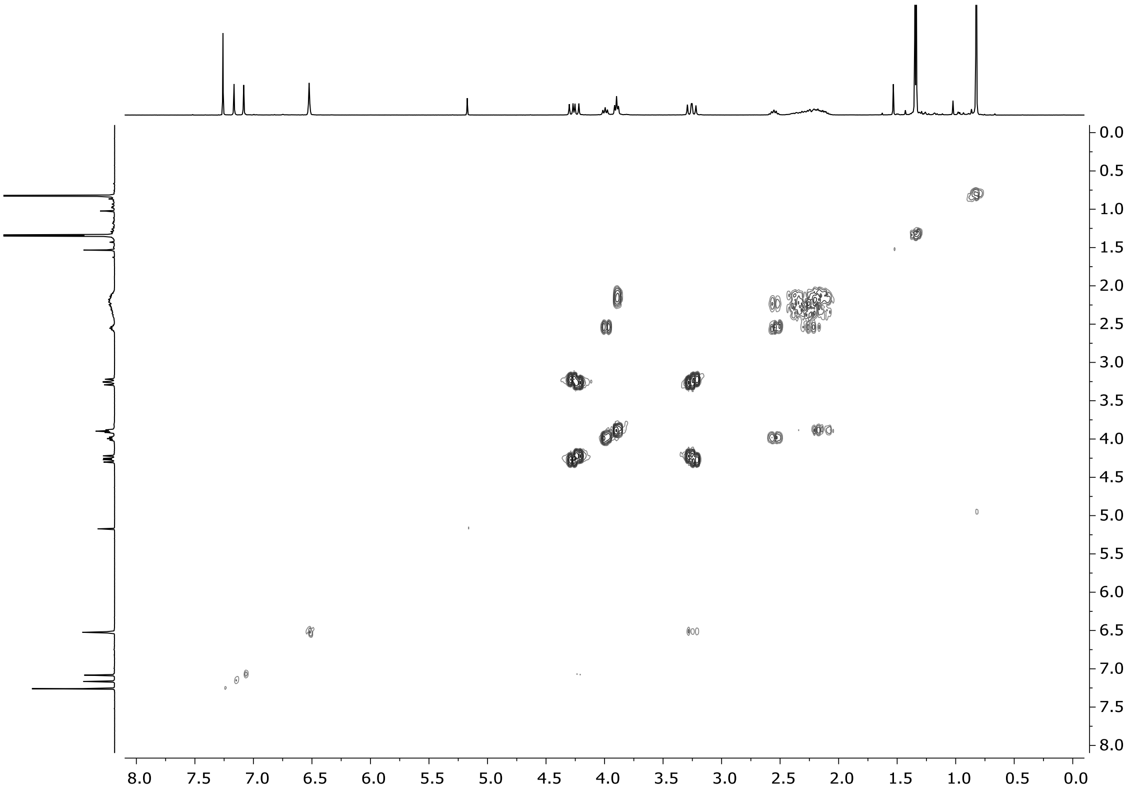

Figure S9. COSY NMR (298K, 400MHz) spectrum of **4** in $CDCl_3$.

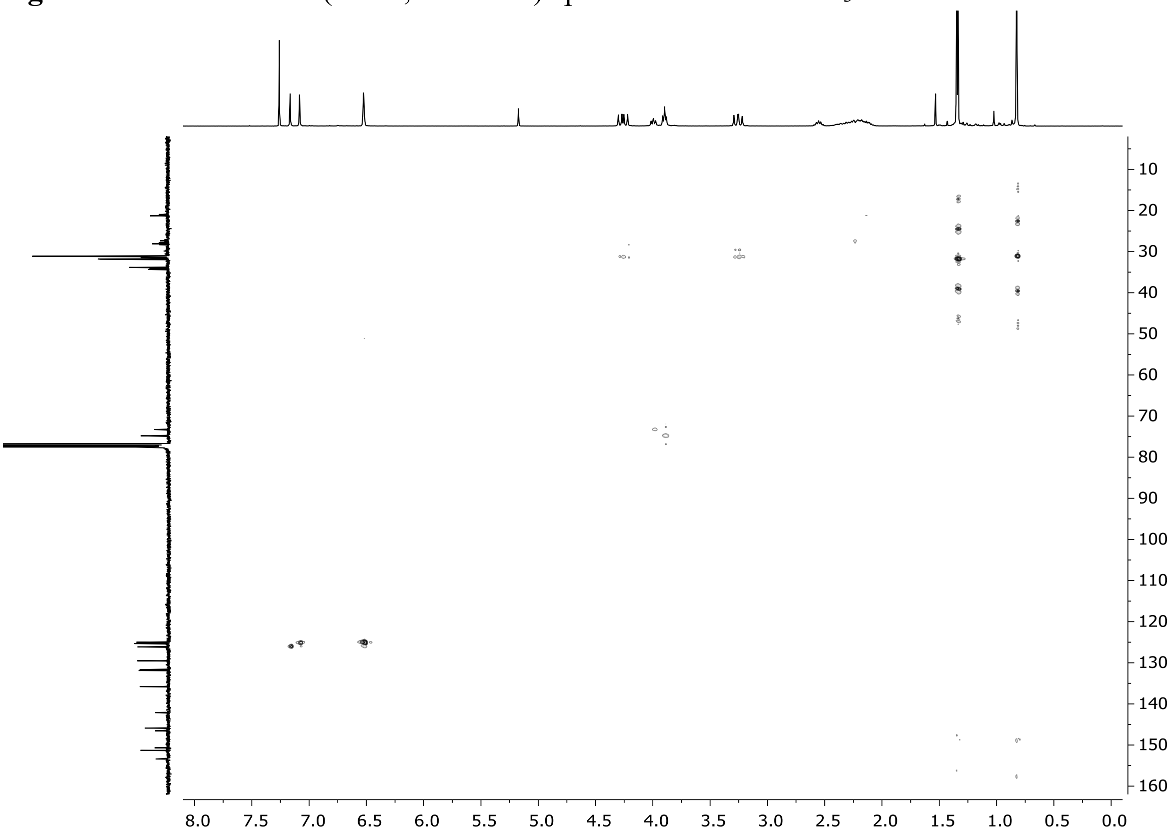

Figure S10. HSQC NMR (298K, 400MHz) spectrum of **4** in $CDCl_3$.

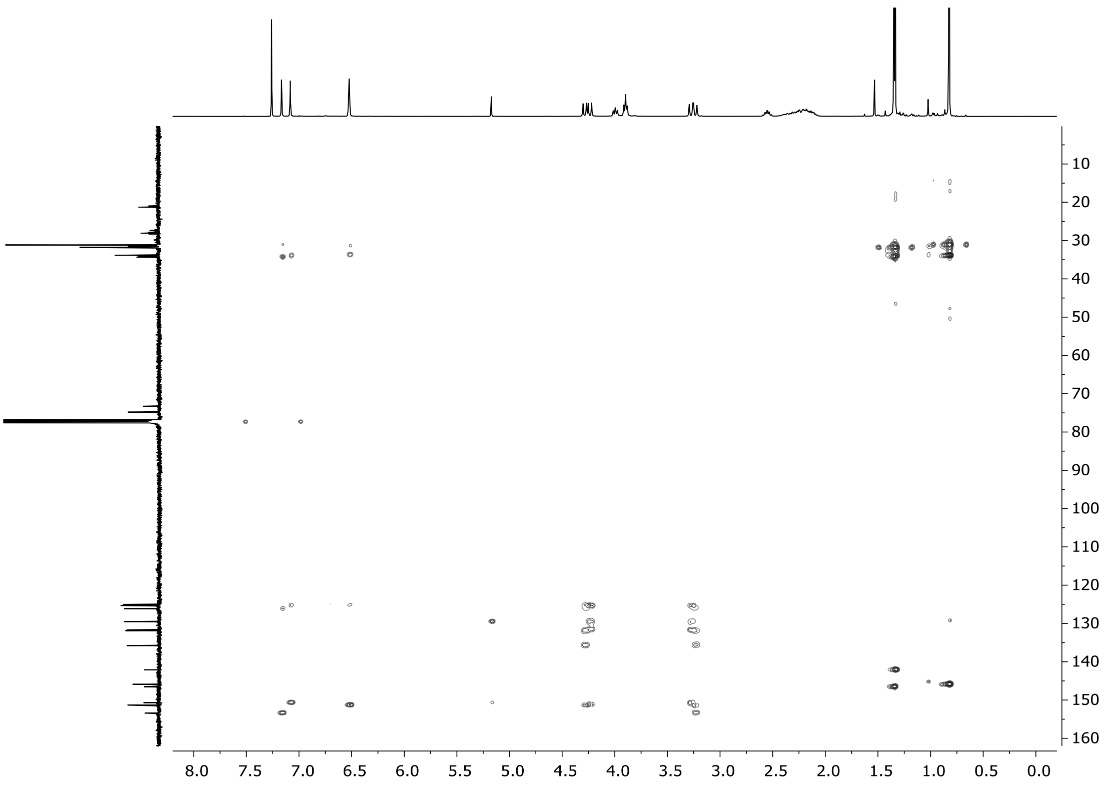

Figure S11. HMBC NMR (298K, 400MHz) spectrum of **4** in $CDCl_3$.

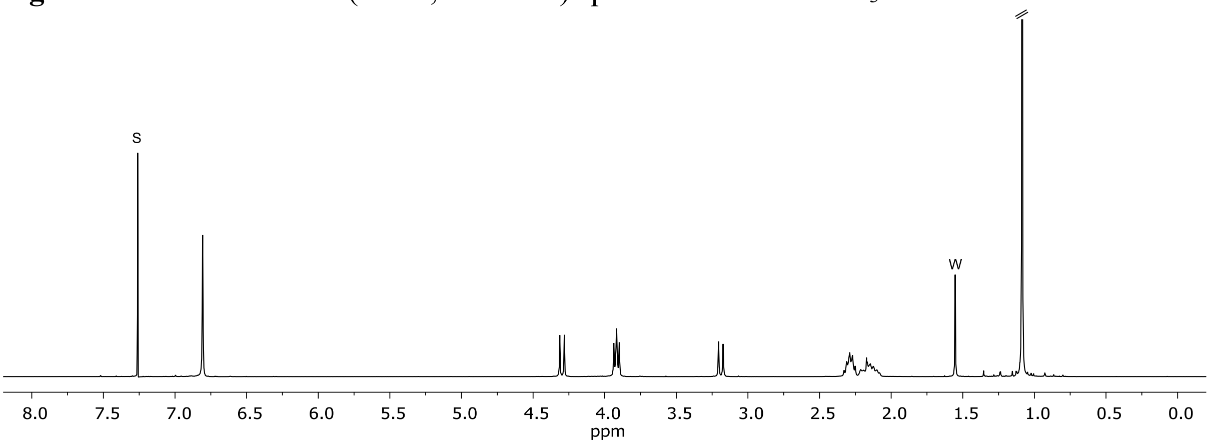

Figure S12. ^{1}H NMR (298K, 400MHz) spectrum of **5** in $CDCl_3$. S = solvent and W = water

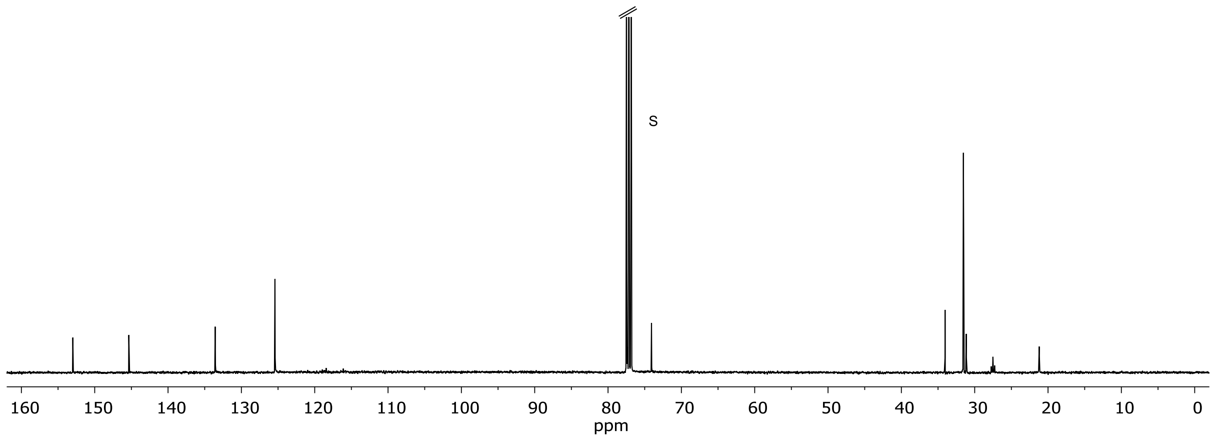

Figure S13. ^{13}C NMR (298K, 100MHz) spectrum of **5** in $CDCl_3$. S = solvent

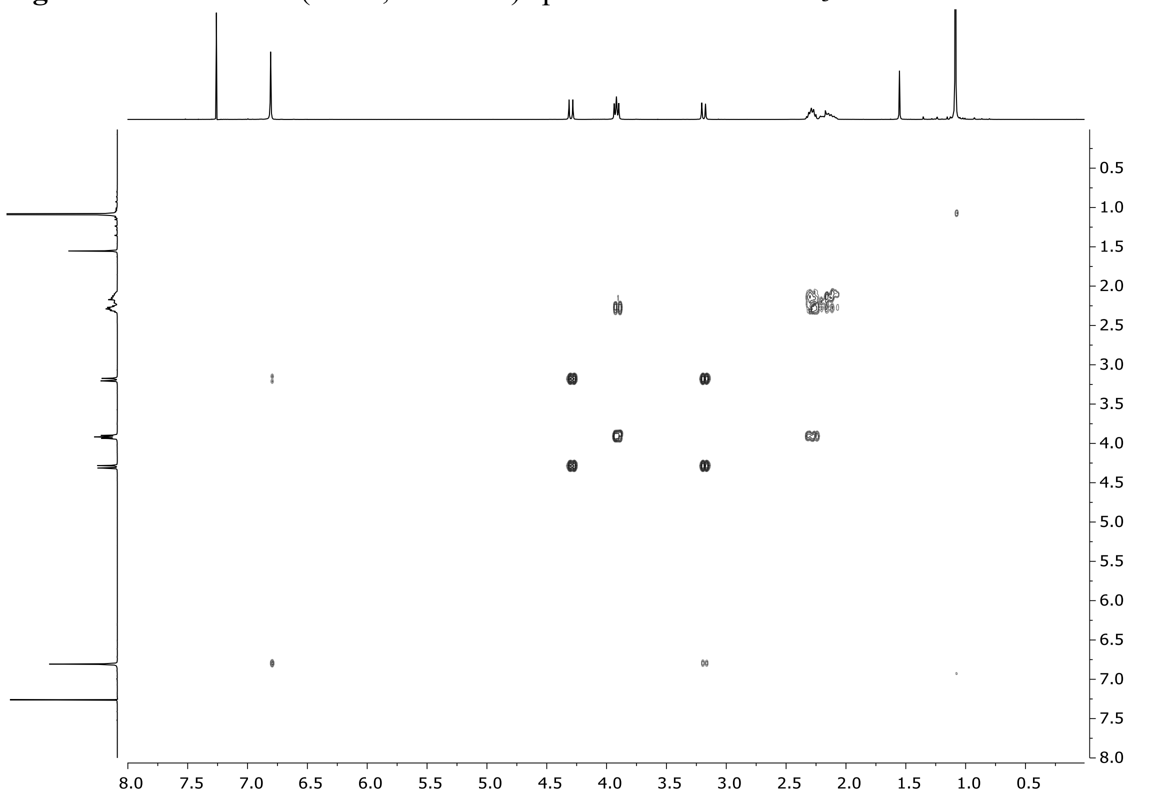

Figure S14. COSY NMR (298K, 400MHz) spectrum of **5** in $CDCl_3$.

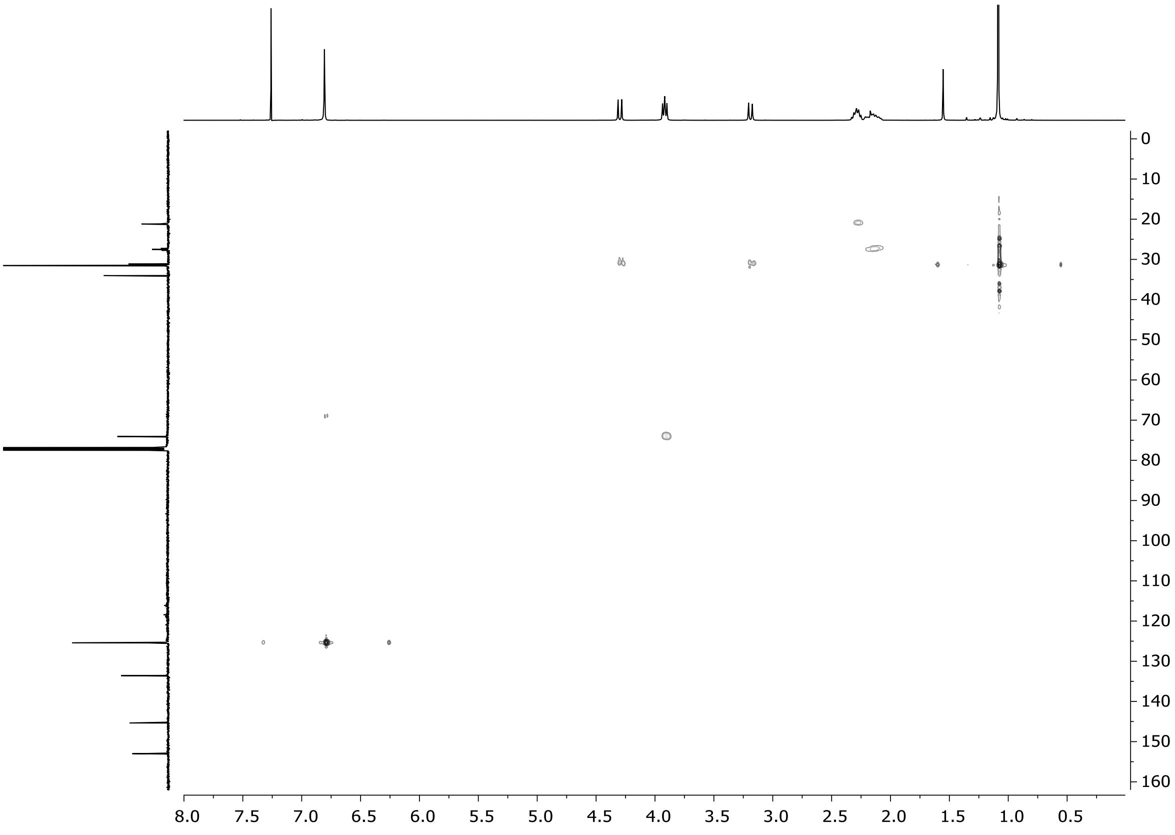

Figure S15. HSQC NMR (298K, 400MHz) spectrum of **5** in $CDCl_3$.

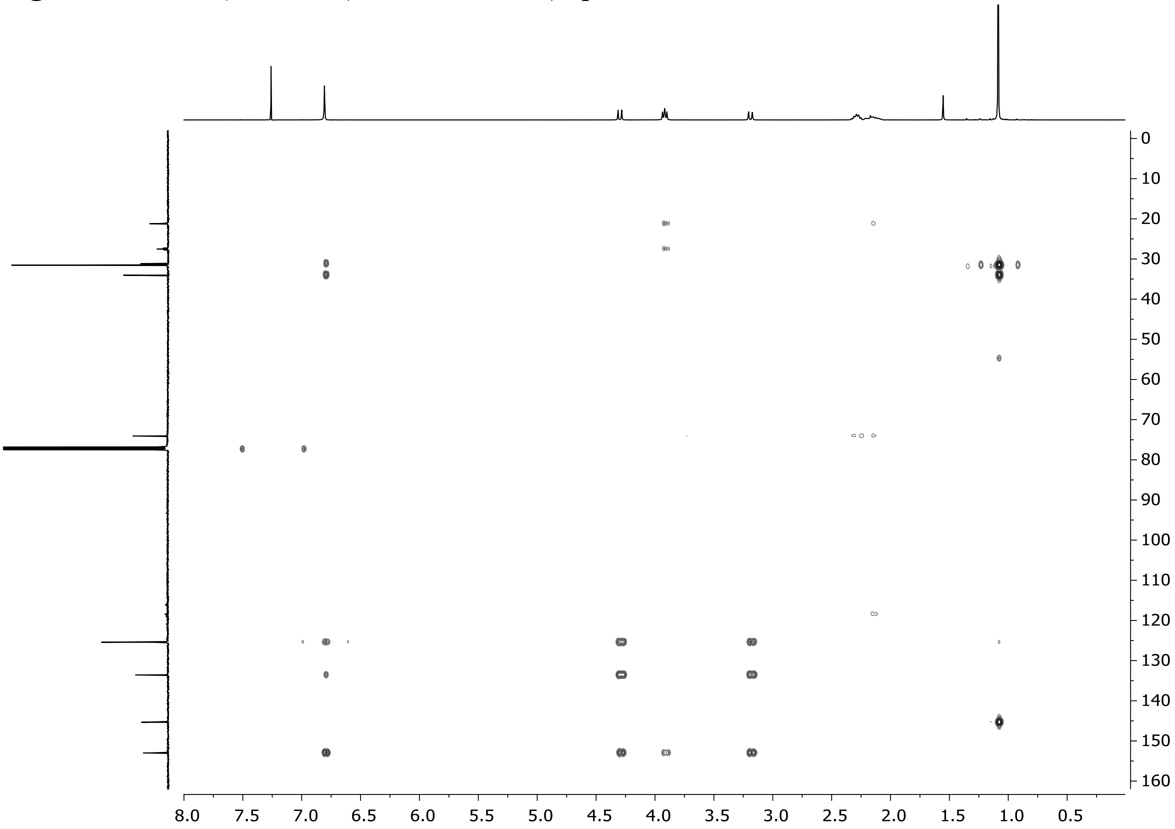

Figure S16. HMBC NMR (298K, 400MHz) spectrum of **5** in $CDCl_3$.

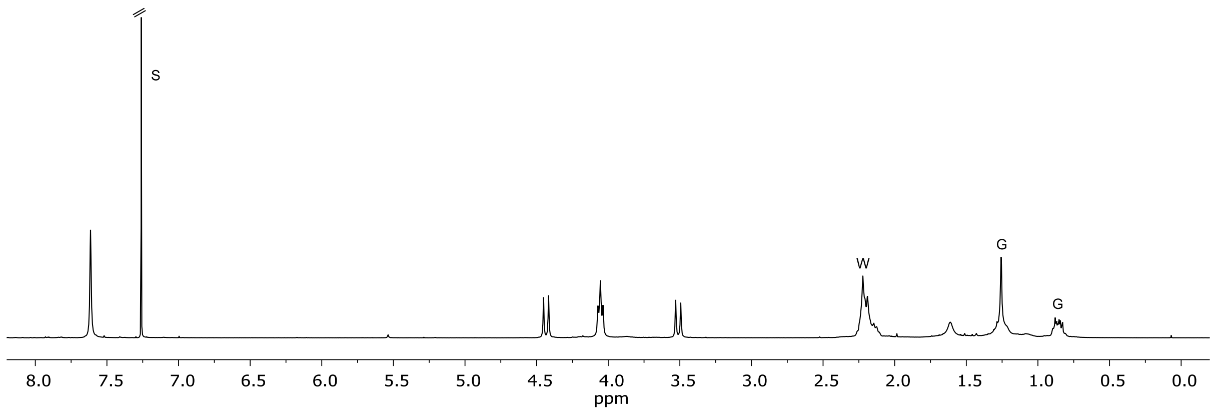

Figure S17. ^{1}H NMR (298K, 400MHz) spectrum of **6** in $CDCl_3$. S = solvent, W = water and G = grease

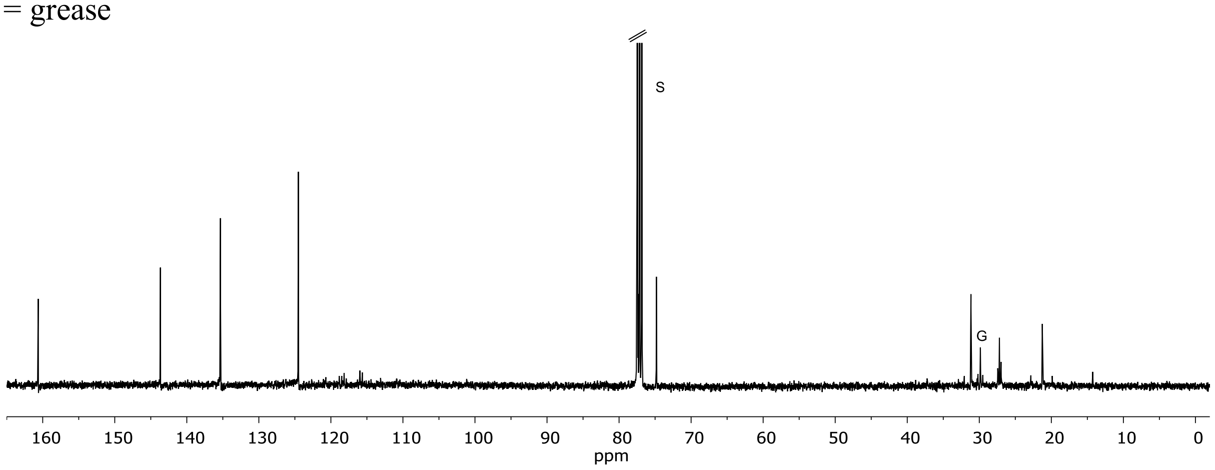

Figure S18. ^{13}C NMR (298K, 100MHz) spectrum of **6** in $CDCl_3$. S = solvent and G = grease

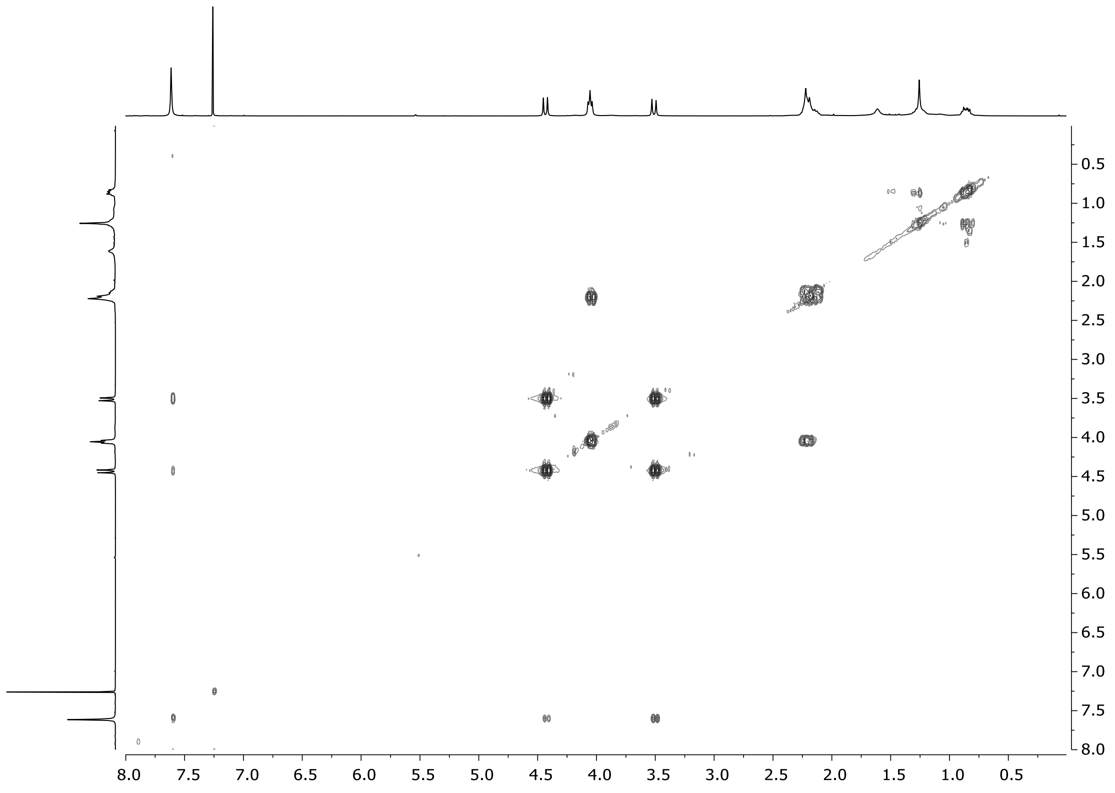

Figure S19. COSY NMR (298K, 400MHz) spectrum of **6** in $CDCl_3$.

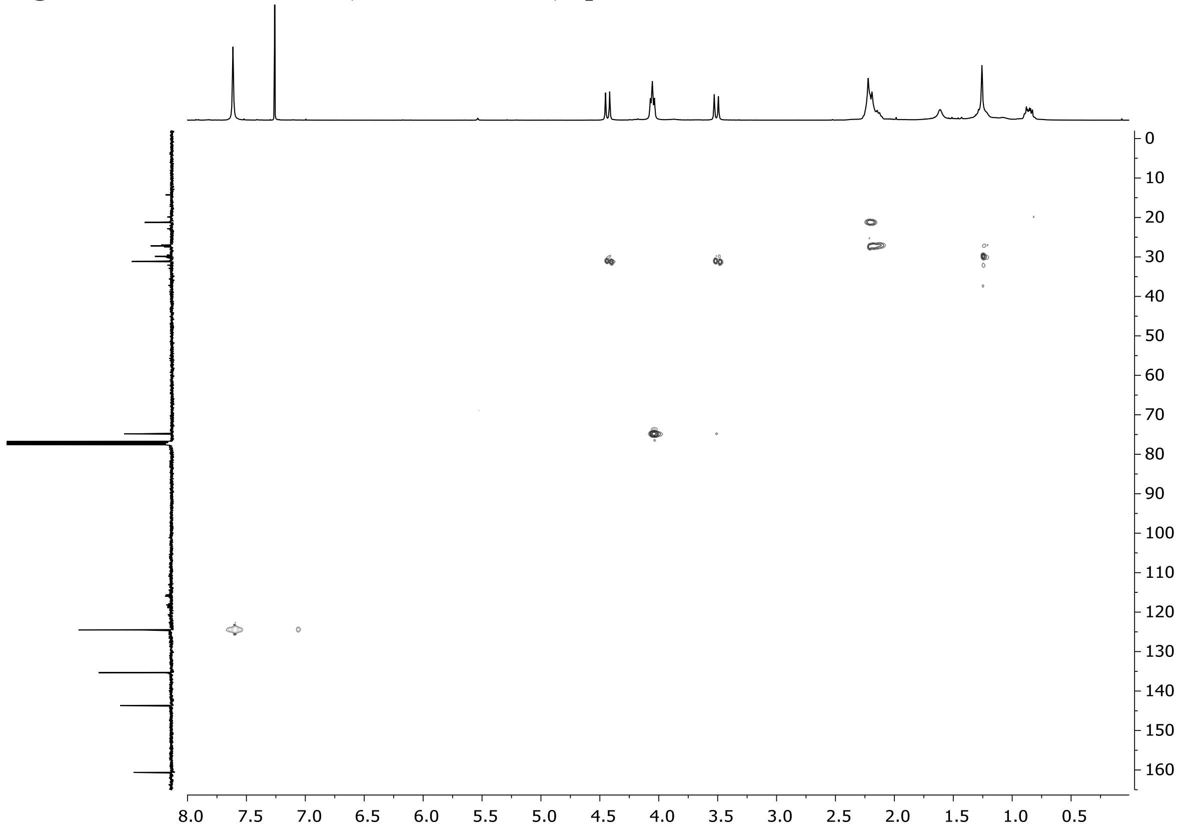

Figure S20. HSQC NMR (298K, 400MHz) spectrum of **6** in $CDCl_3$.

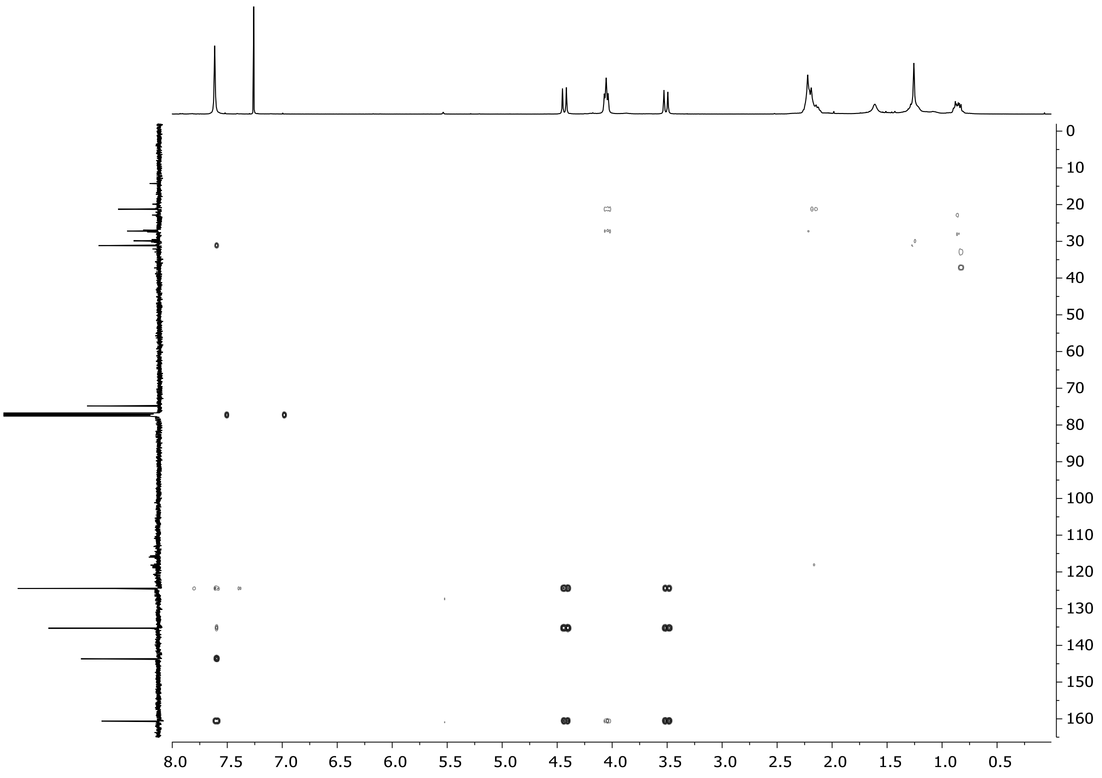

Figure S21. HMBC NMR (298K, 400MHz) spectrum of **6** in $CDCl_3$.

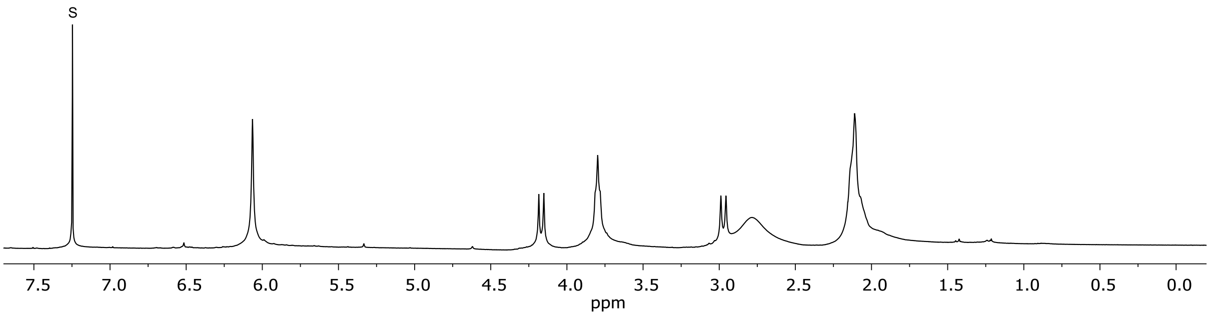

Figure S22. ^{1}H NMR (298K, 400MHz) spectrum of **7** in $CDCl_3$. S = solvent

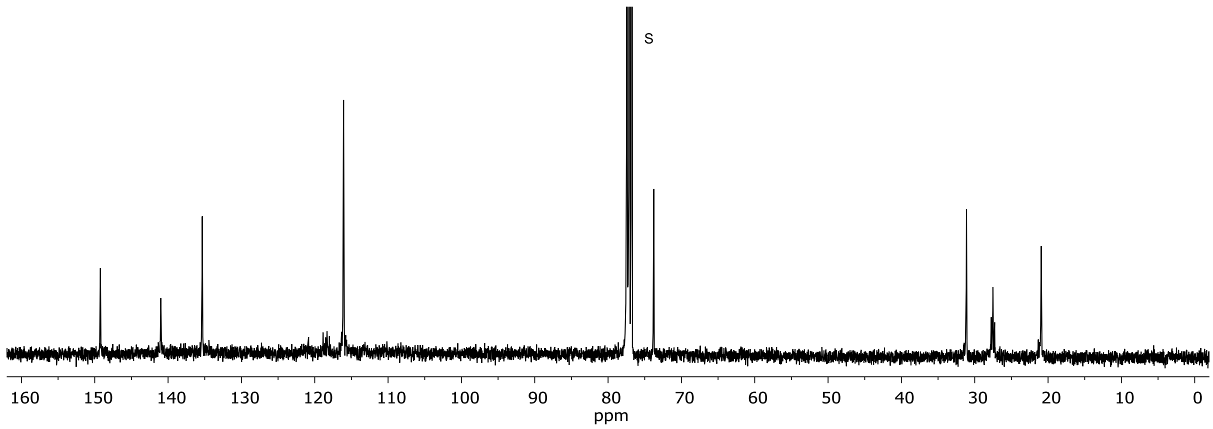

Figure S23. ^{13}C NMR (298K, 100MHz) spectrum of **7** in $CDCl_3$. S = solvent

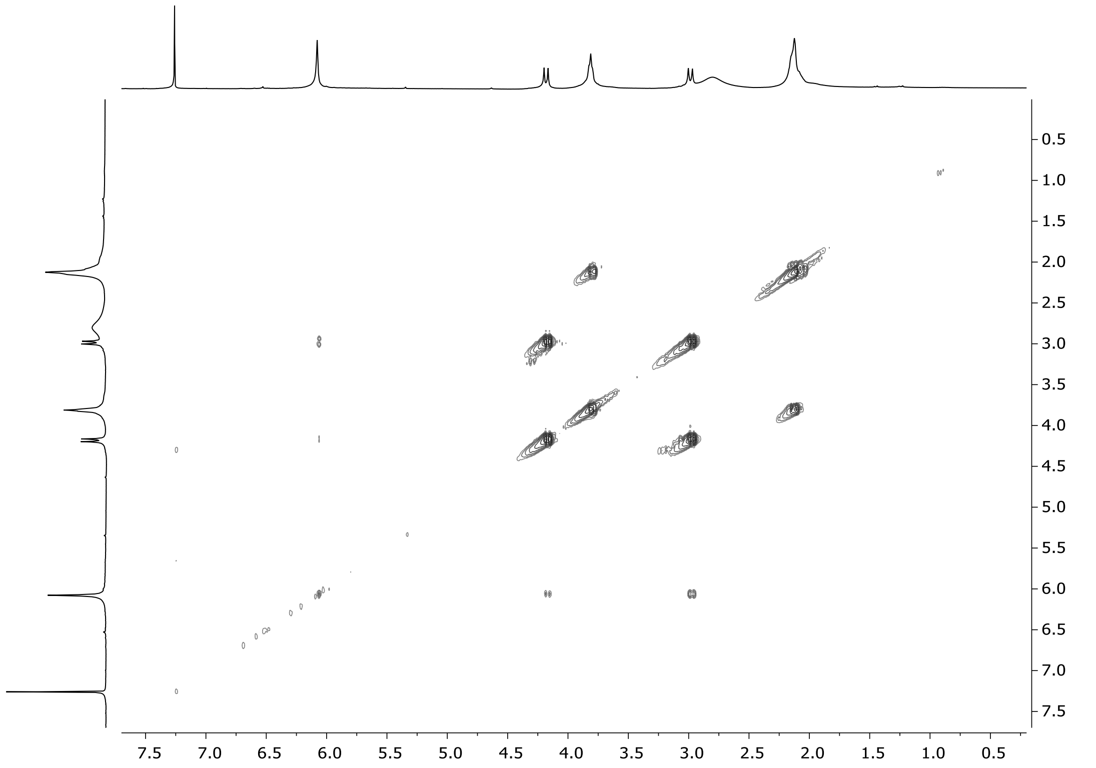

Figure S24. COSY NMR (298K, 400MHz) spectrum of **7** in $CDCl_3$.

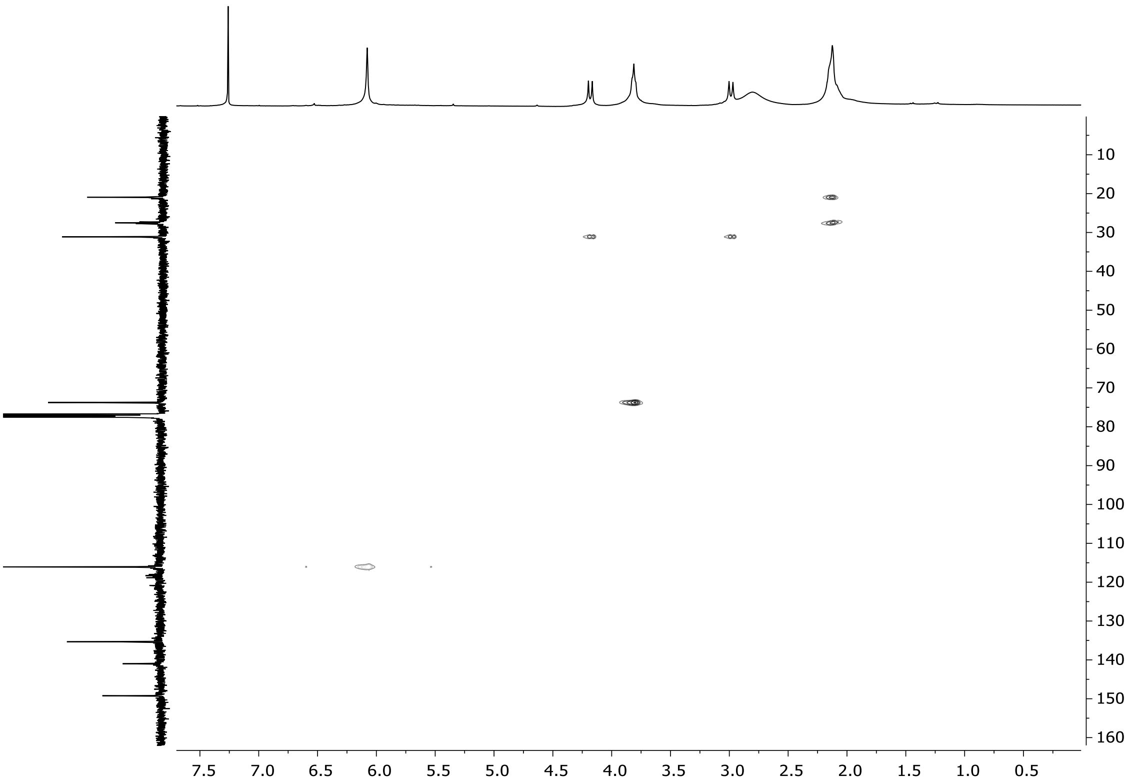

Figure S25. HSQC NMR (298K, 400MHz) spectrum of **7** in $CDCl_3$.

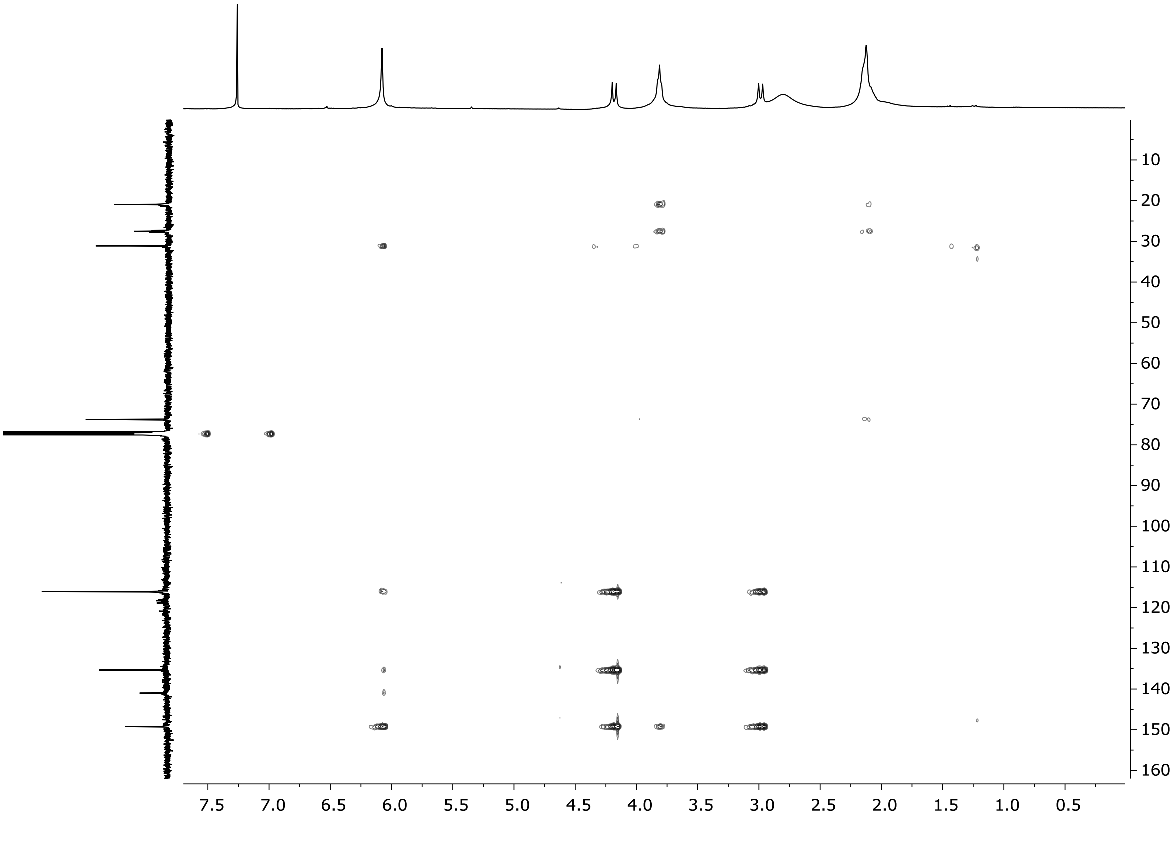

Figure S26. HMBC NMR (298K, 400MHz) spectrum of **7** in $CDCl_3$.

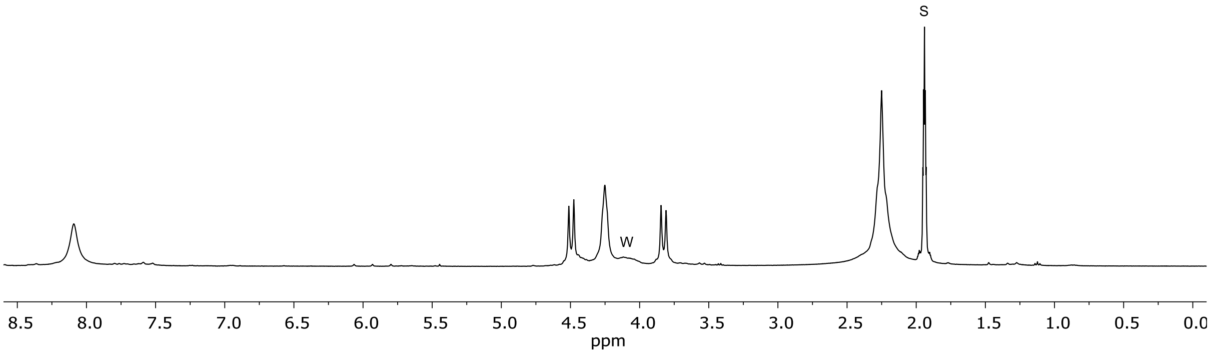

Figure S27. ^{1}H NMR (298K, 400MHz) spectrum of **1** in CD_3CN. S = solvent, W = water.

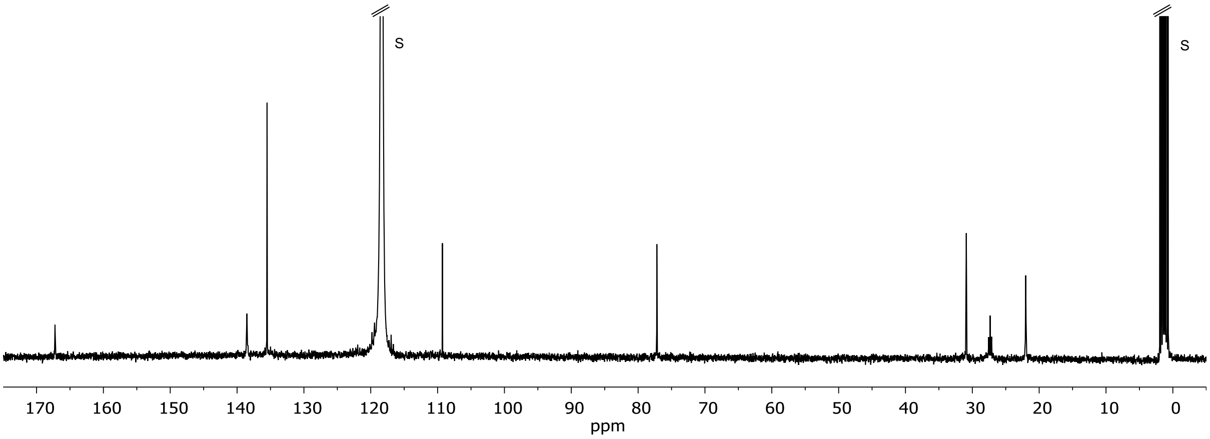

Figure S28. ^{13}C NMR (298K, 100MHz) spectrum of **1** in CD_3CN. S = solvent.

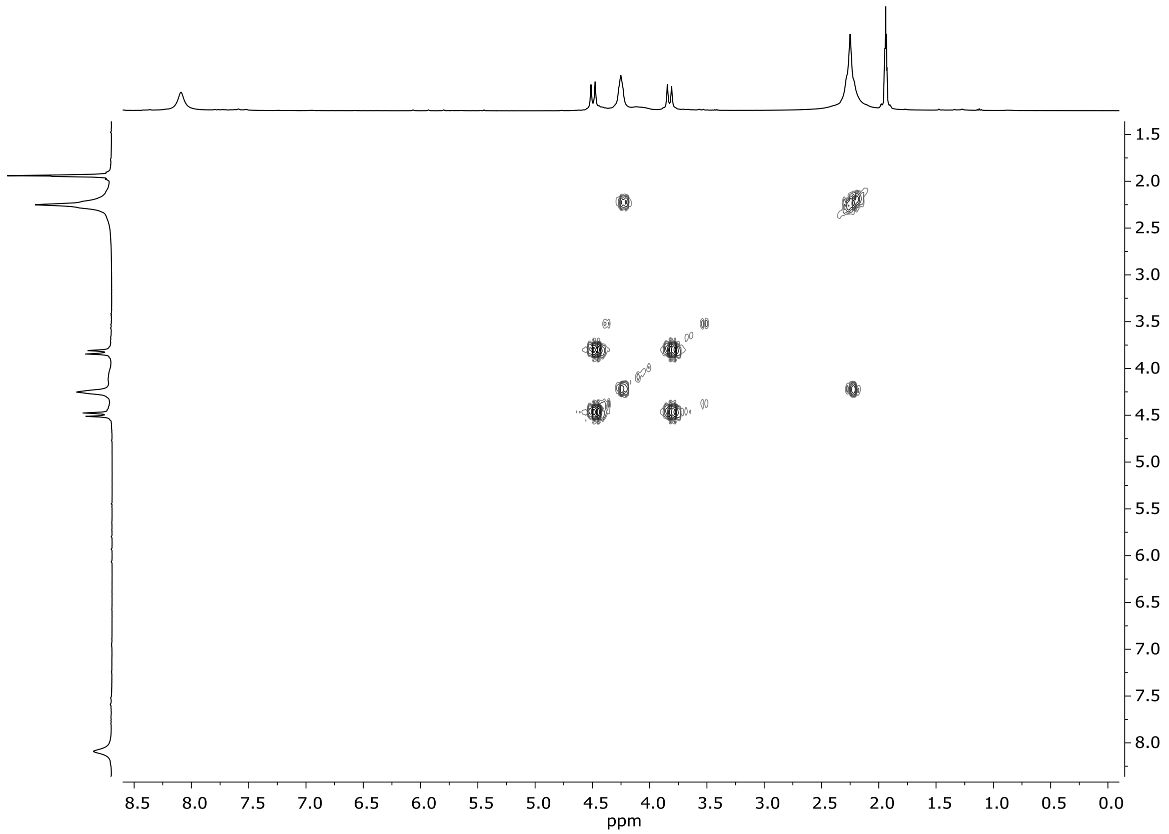

Figure S29. COSY NMR (298K, 400MHz) spectrum of **1** in CD_3CN.

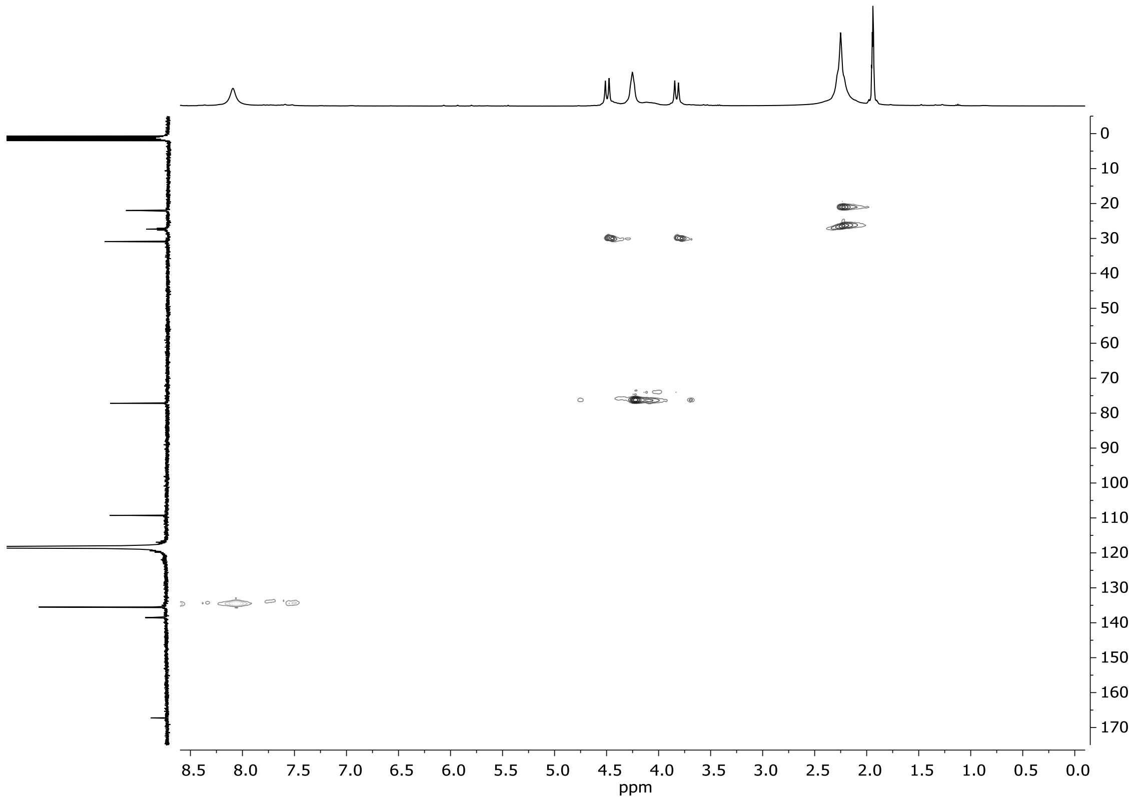

Figure S30. HSQC NMR (298K, 400MHz) spectrum of **1** in CD_3CN.

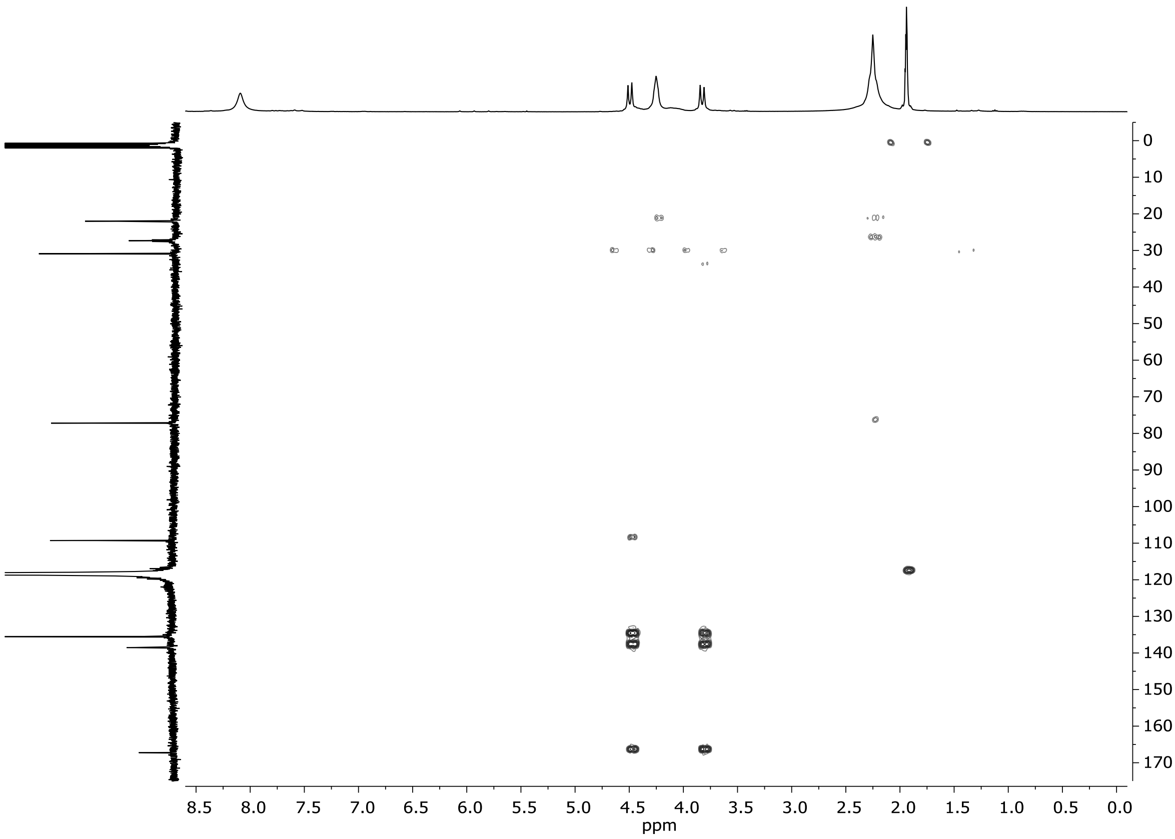

Figure S31. HMBC NMR (298K, 400MHz) spectrum of **1** in CD_3CN.
